# Supplementary material for: Comparative analysis of the complete chloroplast genomes of thirteen Bougainvillea cultivars from South China with implications for their genome structures and phylogenetic relationships
Source: PLoS One. 2024 Sep 11;19(9):e0310091. doi: 10.1371/journal.pone.0310091 (PMC11389920; doi:10.1371/journal.pone.0310091)
Supplement: S1 Fig — First, the B. glabra ‘White Stripe’ chloroplast genome was used as the reference sequence for indels analyses for the other twelve Bougainvillea chloroplast genomes. Second, except B. glabra ‘White Stripe’, the rest 12 Bougainvillea cultivar chloroplast genomes were compared using the chloroplast genome of B. × buttiana ‘Mahara’ as the reference. (A) Total indels statistics. (B) Insertion statistics. (C) Deletion statistics. (DOCX) [file pone.0310091.s001.docx]

**
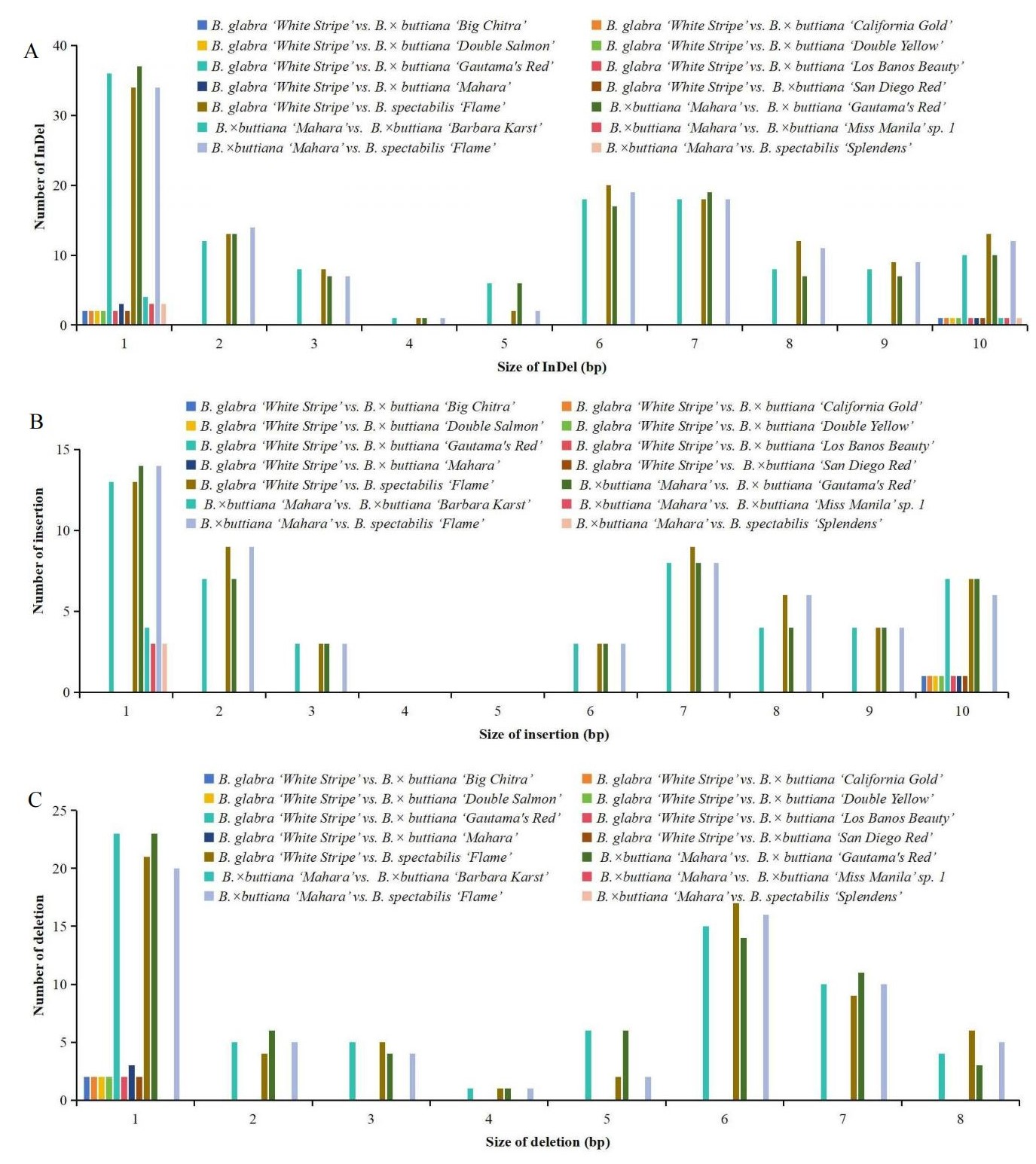
**

**S1 Fig.** Indels statistics of 13 newly sequenced complete chloroplast genomes of the *Bougainvillea* cultivars. First, the *B. glabra ‘*White Stripe’ chloroplast genome was used as the reference sequence for indels analyses for the other twelve *Bougainvillea* chloroplast genomes. Second, except *B. glabra ‘*White Stripe’, the rest 12 *Bougainvillea* cultivar chloroplast genomes were compared using the chloroplast genome of *B.* × *buttiana* ‘Mahara’ as the reference. (A) Total indels statistics. (B) Insertion statistics. (C) Deletion statistics.
