# Supplementary material for: Comparative analysis of the complete chloroplast genomes of thirteen Bougainvillea cultivars from South China with implications for their genome structures and phylogenetic relationships
Source: PLoS One. 2024 Sep 11;19(9):e0310091. doi: 10.1371/journal.pone.0310091 (PMC11389920; doi:10.1371/journal.pone.0310091)
Supplement: S2 Fig — (A) ML tree. (B) BI tree. The 13 newly sequenced Bougainvillea chloroplast genomes identified in this study are shown in bold. (DOCX) [file pone.0310091.s002.docx]

######
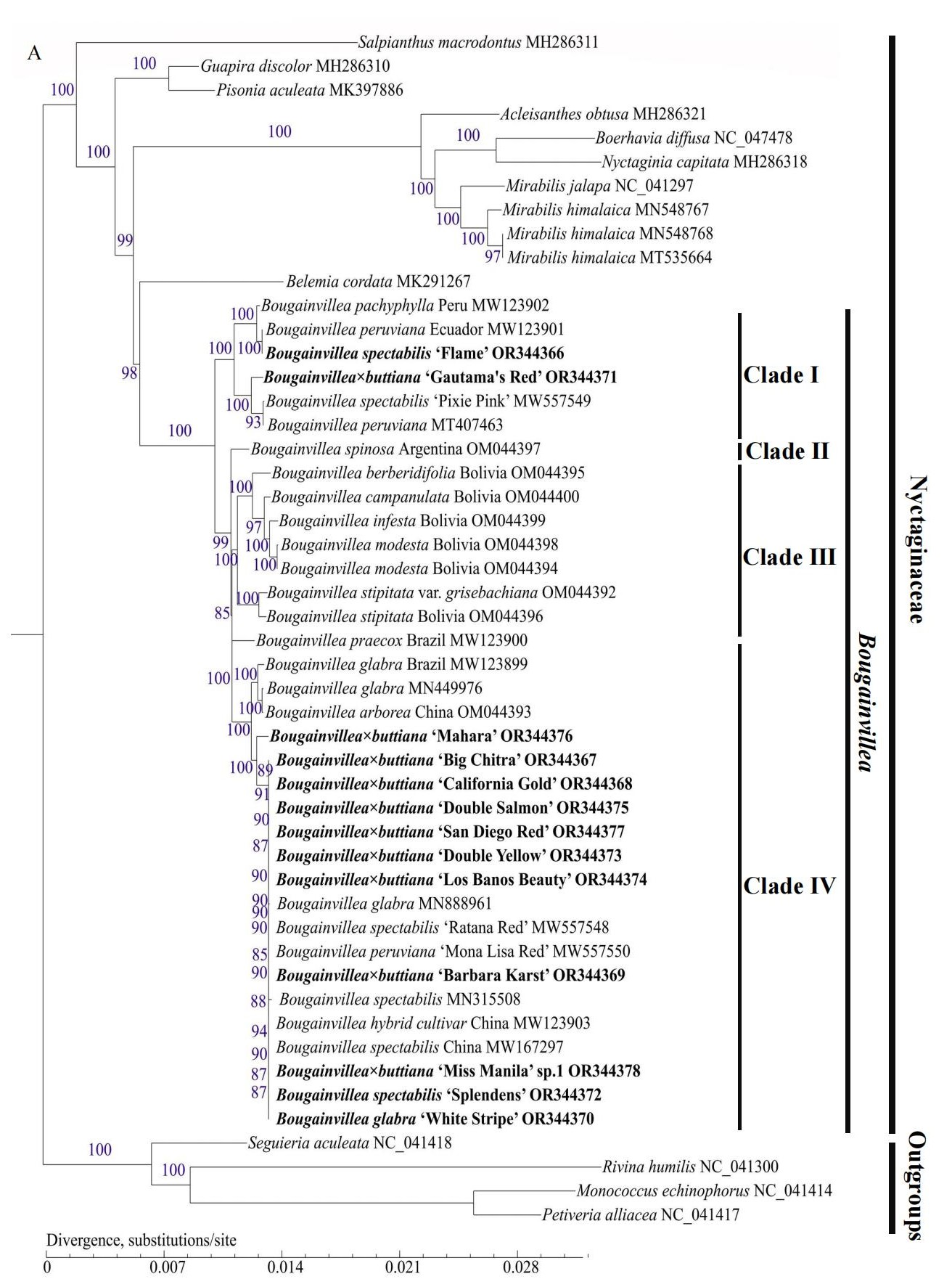


**S2 Fig.** Phylogenetic relationships of Nyctaginaceae species based on protein-coding genes reconstructed using ML and BI methods. (A) ML tree. (B) BI tree. The 13 newly sequenced *Bougainvillea* chloroplast genomes in this study are in bold.

**
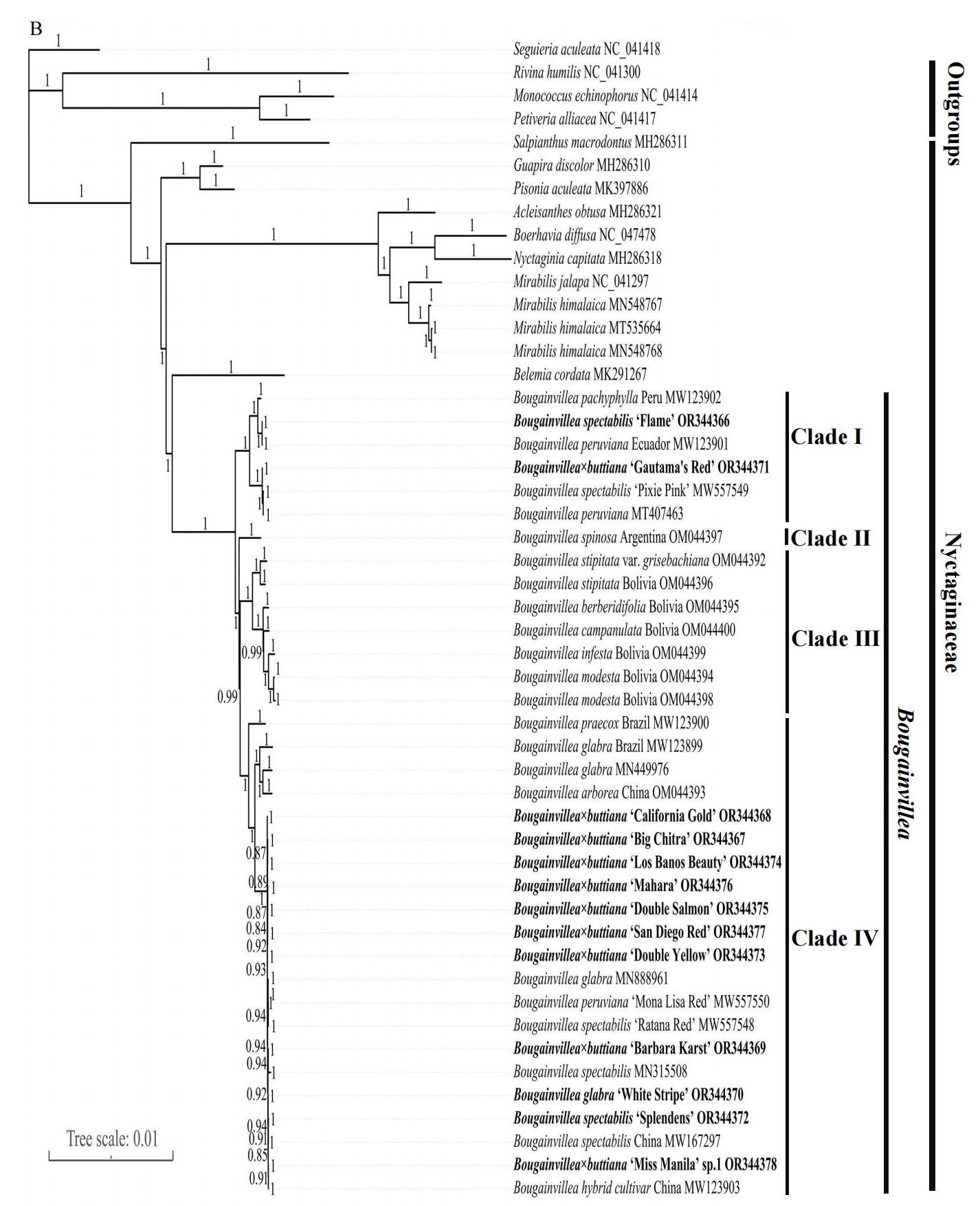
**

**S2 Fig.** continued.
