# Supplementary material for: Comparative analysis of the complete chloroplast genomes of thirteen Bougainvillea cultivars from South China with implications for their genome structures and phylogenetic relationships
Source: PLoS One. 2024 Sep 11;19(9):e0310091. doi: 10.1371/journal.pone.0310091 (PMC11389920; doi:10.1371/journal.pone.0310091)
Supplement: S1 Table — (DOCX) [file pone.0310091.s003.docx]

**S1 Table.**Information on the 13 *Bougainvillea* cultivars.

| **Cultivars** | Date of collection | Collector numbers | Store sheet  code | Insert size (bp) | Raw data (Gb) | Clean data (Gb) | Clean data Q20 (%) | Clean data Q30 (%) | Clean data GC (%) |
| --- | --- | --- | --- | --- | --- | --- | --- | --- | --- |
| *Bougainvillea* × buttiana ‘Mahara’ | 7 April, 2023 | X. Y. Wu and D. M. Li, 79 | B202379 | 450 | 6.082 | 6.070 | 97.76 | 93.05 | 38.22 |
| B. × buttiana ‘Gautama's Red’ | 7 April, 2023 | X. Y. Wu and D. M. Li, 42 | B202342 | 450 | 6.018 | 6.007 | 97.66 | 92.75 | 37.55 |
| B. × buttiana ‘California Gold’ | 7 April, 2023 | X. Y. Wu and D. M. Li, 13 | B202313 | 450 | 5.975 | 5.964 | 97.81 | 93.22 | 37.71 |
| B. × buttiana ‘Double Salmon’ | 7 April, 2023 | X. Y. Wu and D. M. Li, 77 | B202377 | 450 | 7.357 | 7.342 | 97.50 | 92.28 | 38.04 |
| B. × buttiana ‘Double Yellow’ | 7 April, 2023 | X. Y. Wu and D. M. Li, 75 | B202375 | 450 | 6.541 | 6.528 | 97.59 | 92.53 | 37.80 |
| B. × buttiana ‘Big Chitra’ | 7 April, 2023 | X. Y. Wu and D. M. Li, 8 | B202308 | 450 | 7.061 | 7.049 | 97.87 | 93.39 | 37.73 |
| B. × buttiana ‘Los Banos Beauty’ | 7 April, 2023 | X. Y. Wu and D. M. Li, 76 | B202376 | 450 | 6.528 | 6.513 | 97.58 | 92.50 | 38.30 |
| B. glabra ‘White Stripe’ | 7 April, 2023 | X. Y. Wu and D. M. Li, 19 | B202319 | 450 | 5.875 | 5.865 | 97.74 | 93.00 | 38.24 |
| B. spectabilis ‘Flame’ | 7 April, 2023 | X. Y. Wu and D. M. Li, No.7 | B2023No.7 | 450 | 6.641 | 6.629 | 98.98 | 95.85 | 36.38 |
| B. spectabilis ‘Splendens’ | 7 April, 2023 | X. Y. Wu and D. M. Li, 58 | B202358 | 450 | 5.611 | 5.602 | 97.92 | 93.53 | 37.93 |
| 1. × buttiana   ‘Barbara Karst’ | 7 April, 2023 | X. Y. Wu and D. M. Li, 16 | B202316 | 450 | 7.275 | 7.259 | 97.60 | 92.56 | 37.81 |
| B.× buttiana ‘San Diego Red’ | 7 April, 2023 | X. Y. Wu and D. M. Li, 103 | B2023103 | 450 | 6.960 | 6.947 | 97.76 | 93.05 | 37.69 |
| B.× buttiana ‘Miss Manila’ sp.1 | 7 April, 2023 | X. Y. Wu and D. M. Li, No.1W | B2023No.1W | 450 | 8.613 | 8.599 | 99.04 | 96.06 | 36.72 |
