# Supplementary material for: Comparative analysis of the complete chloroplast genomes of thirteen Bougainvillea cultivars from South China with implications for their genome structures and phylogenetic relationships
Source: PLoS One. 2024 Sep 11;19(9):e0310091. doi: 10.1371/journal.pone.0310091 (PMC11389920; doi:10.1371/journal.pone.0310091)
Supplement: S2 Table — (DOCX) [file pone.0310091.s004.docx]

**S2 Table.** The 46 complete chloroplast genomes in family Nyctaginaceae used for selective pressure and phylogenetic relathionships.

| Number | Species name | GenBank number |
| --- | --- | --- |
| 1 | *Bougainvillea spectabilis* | MN315508 |
| 2 | *Bougainvillea glabra* | MN449976 |
| 3 | *Bougainvillea glabra* | MN888961 |
| 4 | *Bougainvillea peruviana* | MT407463 |
| 5 | *Bougainvillea glabra* Brazil | MW123899 |
| 6 | *Bougainvillea praecox* Brazil | MW123900 |
| 7 | *Bougainvillea peruviana* Ecuador | MW123901 |
| 8 | *Bougainvillea pachyphylla* Peru | MW123902 |
| 9 | *Bougainvillea* hybrid cultivar China | MW123903 |
| 10 | *Bougainvillea spectabilis* China | MW167297 |
| 11 | *Bougainvillea stipitata* var. *grisebachiana* | OM044392 |
| 12 | *Bougainvillea arborea* China | OM044393 |
| 13 | *Bougainvillea modesta* Bolivia | OM044394 |
| 14 | *Bougainvillea berberidifolia* Bolivia | OM044395 |
| 15 | *Bougainvillea stipitata* Bolivia | OM044396 |
| 16 | *Bougainvillea spinosa* Argentina | OM044397 |
| 17 | *Bougainvillea modesta* Bolivia | OM044398 |
| 18 | *Bougainvillea infesta* Bolivia | OM044399 |
| 19 | *Bougainvillea campanulata* Bolivia | OM044400 |
| 20 | *Bougainvillea spectabilis‘*Ratana Red*’* | MW557548 |
| 21 | *Bougainvillea spectabilis* *‘*Pixie Pink*’* | MW557549 |
| 22 | *Bougainvillea peruviana* ‘Mona Lisa Red’ | MW557550 |
| 23 | ***Bougainvillea*×*buttiana* ‘Mahara’** | **OR344376** |
| 24 | ***Bougainvillea*×*buttiana* ‘Gautama's Red’** | **OR344371** |
| 25 | ***Bougainvillea*×*buttiana* ‘California Gold’** | **OR344368** |
| 26 | ***Bougainvillea*×*buttiana* ‘Double Salmon’** | **OR344375** |
| 27 | ***Bougainvillea*×*buttiana* ‘Double Yellow’** | **OR344373** |
| 28 | ***Bougainvillea*×*buttiana* ‘Big Chitra’** | **OR344367** |
| 29 | ***Bougainvillea glabra ‘*White Stripe’** | **OR344370** |
| 30 | ***Bougainvillea*×*buttiana* ‘Los Banos Beauty’** | **OR344374** |
| 31 | ***Bougainvillea spectabilis* ‘Flame’** | **OR344366** |
| 32 | ***Bougainvillea spectabilis* ‘Splendens’** | **OR344372** |
| 33 | ***Bougainvillea* ‘Barbara Karst’** | **OR344369** |
| 34 | ***Bougainvillea* ‘San Diego Red’** | **OR344377** |
| 35 | ***Bougainvillea* sp.1** | **OR344378** |
| 36 | *Nyctaginia capitata* | MH286318 |
| 37 | *Mirabilis jalapa* | NC_041297 |
| 38 | *Acleisanthes obtusa* | MH286321 |
| 39 | *Guapira discolor* | MH286310 |
| 40 | *Pisonia aculeata* | MK397886 |
| 41 | *Salpianthus macrodontus* | MH286311 |
| 42 | *Mirabilis himalaica* | MT535664 |
| 43 | *Belemia cordata* | MK291267 |
| 44 | *Mirabilis himalaica* | MN548767 |
| 45 | *Mirabilis himalaica* | MN548768 |
| 46 | *Boerhavia diffusa* | NC_047478 |

Note: The cultivars in bold are sequenced in this study.
