# Supplementary material for: Comparative analysis of the complete chloroplast genomes of thirteen Bougainvillea cultivars from South China with implications for their genome structures and phylogenetic relationships
Source: PLoS One. 2024 Sep 11;19(9):e0310091. doi: 10.1371/journal.pone.0310091 (PMC11389920; doi:10.1371/journal.pone.0310091)
Supplement: Attachment — Submitted filename: reviewer response.6.11.pdf [file pone.0310091.s014.pdf]

Reviewer #1: It is highly appreciated that the authors have made substantial efforts in addressing most of the minor issues and improving comprehensibility of the manuscript. However, the major issues were not settled, particularly the cultivar nomenclature and the clustering of clades. It is extremely irresponsible that the authors claimed all the cultivars unregistered only because of their inaccessiblity to the website of Bougainvillea Society of India. Some of the studied cultivars were registered while some are not. This act is seriously misleading to the readers since they claimed some legitimate cultivar epithets as illegitimate. The authors should try their best to reach to the pieces of information regardless of any format (e.g. books, printed literatures and any electronic means), but not just giving up and leave an incorrect and irrespinsible statement.

Response: We removed the statement. The 12 *Bougainvillea* cultivars included *B.* × *buttiana* 'Mahara' , *B.* × *buttiana* 'California Gold', *B.* × *buttiana* 'Double Yellow', *B.* × *buttiana* 'Los Banos Beauty', *B.* × *buttiana* 'San Diego Red', *B.* ×*buttiana* 'Barbara Karst', *B. spectabilis* 'Flame', *B. spectabilis* 'Splendens', *B.* × *buttiana* 'Gautama's Red', *B.* × *buttiana* 'Double Salmon', *B.* × *buttiana* 'Big Chitra', and *B. glabra* 'White Stripe'. Their names came from following information:

Liu Y, Ruan L, Zhou H, Yu M. Cultivar classification of *Bougainvillea*. China Forestry Press, Beijing, China, 2020

Sun L. Molecular identification of cultivars and transcriptome analysis of bracts in *Bougainvillea*. Ph. D. Chinese Academy of Forestry, Beijing, China, 2019, pp38-39

From the description of Sun in 2019, *B.*× *buttiana* 'San Diego Red' and *B.* ×*buttiana* 'Barbara Karst' were the correct names for *Bougainvillea* 'San Diego Red' and *Bougainvillea* 'Barbara Karst', respectively. We used *B.* × *buttiana* 'San Diego Red' and *B.* ×*buttiana* 'Barbara Karst' in the whole text instead of *B.* 'San Diego Red' and *B.* 'Barbara Karst', respectively.

Because the thornless mutant was a variety from *Bougainvillea* × *buttiana* 'Miss Manila' and the author He-Fa Wang hasn't yet got certificate of registered commercial name for the mutant, we gave the mutant with the name of *Bougainvillea* × *buttiana* 'Miss Manila' sp.1 in the text.

For the clustering of Bougainvillea spinosa, the authors obviously showed their wrong concept on monophyly. The claims of the authors (Bougainvillea spinosa was in either clade I or II) is very misleading to the readers outside the field of phylogeny.

Response: Thanks for your opinion on the clustering of *Bougainvillea spinosa*.

In Bautista et al. (2022), *B. spinosa* was sister to clade II or wild clade *Bougainvillea* in that study. However, Lin et al. (2023) clustered the *B. spinosa* into clade 3, which was the same clade in our study, clade I.

Bautista MAC, Zheng Y, Boufford DE, Hu Z, Deng Y, Chen T. Phylogeny and taxonomic synopsis of the genus Bougainvillea (Nyctaginaceae). Plants (Basel). 2022; 11, 1700.

https://doi.org/10.3390/plants11131700 PMID: 35807654
Lin X, Lee SY, Ni J, Zhang X, Hu X, Zou P, Wang W, Liu G. Comparative analyses of chloroplast genome provide effective molecular markers for species and cultivar identification in *Bougainvillea*. Int J Mol Sci. 2023; 24(20),15138. https://doi.org/10.3390/ijms242015138. PMID: 37894819

We revised the section Phylogenetic relationships in the *Bougainvillea* genus as following:

The 35 *Bougainvillea* individuals analyzed were divided into four clades, namely, Clades I, II, III, and IV, with strongly supported values (BS = 85–100% for the ML trees and PP = 0.99–1 for the BI trees) (Fig 9, S2 Fig). Two cultivars, *B.* × *buttiana* 'Gautama's Red' and *B. spectabilis* 'Flame', were clustered into clade I, and the other 11 cultivars, including *B.* × *buttiana* 'Mahara', *B.* × *buttiana* 'California Gold', *B.* × *buttiana* 'Double Salmon', *B.* × *buttiana* 'Double Yellow', *B.* × *buttiana* 'Los Banos Beauty', *B.* × *buttiana* 'Big Chitra', *B.* × *buttiana* 'Barbara Karst', *B. glabra* 'White Stripe', *B. spectabilis* 'Splendens', *B.* × *buttiana* 'San Diego Red', and *B.* × *buttiana* 'Miss Manila' sp. 1, were clustered into clade IV (Fig 9, S2 Fig). In Clade I, *B. spectabilis* 'Flame' was sister to *B. peruviana* MW123901 and then formed a strong sister cluster to *B. pachyphylla*, both based on chloroplast genome sequences and protein-coding genes (Fig 9, S2 Fig). However, the position of *B.* × *buttiana* 'Gautama's Red' in the ML tree constructed from chloroplast genome sequences differed from those in the other three phylogenetic trees in this study. For the former, *B.* × *buttiana* 'Gautama's Red' was sister to *B. spectabilis* 'Pixie Pink' and then clustered with *B. peruviana* MT407463 with strong support (BS = 90%) (Fig 9A). For the latter, *B. spectabilis* 'Pixie Pink' was sister to *B. peruviana* MT407463 and then clustered with *B.* × *buttiana* 'Gautama's Red' with strong support (BS = 93–100%, and PP = 0.93–1) (Fig 9B, S2 Fig). In Clade II, it only contained *B. spinosa* (Fig 9, S2 Fig). In Clade III, there were 7 individuals of wild species, including *B. campanulata*, *B. berberidifolia*, *B. infesta*, *B. modesta* OM44398, *B. modesta* OM044396, *B. stipitata*, and *B. stipitata var. grisebachiana*. In Clade IV, in the ML tree based on the chloroplast genome sequences, *B.* × *buttiana* 'Mahara', *B.* × *buttiana* 'California Gold', *B.* × *buttiana* 'Double Salmon', *B.* × *buttiana* 'Double Yellow', *B.* × *buttiana* 'Los Banos Beauty', *B.* × *buttiana* 'Big Chitra', *B.* × *buttiana* 'San Diego Red', *B. spectabilis* 'Ratana Red', *B. glabra*

MN888961, and *B. peruviana* 'Mona Lisa Red' were clustered together in one cluster with strong support (BS = 88–92%). *B. × buttiana* 'Barbara Karst', *B. glabra* 'White Stripe', *B. spectabilis* 'Splendens', *B. × buttiana* 'Miss Manila' sp. 1, *B. spectabilis* MN315508, *B. spectabilis* China MW167297, and *B.* hybrid cultivar MW123903 were clustered together in another cluster with strong support (BS =88–95%) (Fig 9A). However, in the ML tree based on protein-coding genes, *B. × buttiana* 'Mahara' was sister to the other cultivars in Clade IV with strong support (BS = 100%) (S2A Fig). In both BI trees, *B. × buttiana* 'Mahara', *B. × buttiana* 'California Gold', *B. × buttiana* 'Double Salmon', *B. × buttiana* 'Double Yellow', *B. × buttiana* 'Los Banos Beauty', *B. × buttiana* 'Big Chitra', *B. × buttiana* 'San Diego Red', *B. spectabilis* 'Ratana Red', *B. glabra*, *B. peruviana* 'Mona Lisa Red', *B. × buttiana* 'Barbara Karst', *B. glabra* 'White Stripe', *B. spectabilis* 'Splendens', *B. × buttiana* 'Miss Manila' sp. 1, *B. spectabilis* MN315508, *B. spectabilis* MW167297, and *B.* hybrid cultivar MW123903 were clustered together in one cluster in Clade IV with moderate to strong support (PP = 0.84–1) (Fig 9B, S2B Fig). In the four phylogenetic trees, Clades III and IV were clustered together, forming a cluster with strong support (BS =85–99%, and PP = 0.99–1); then the cluster, Clade II, and Clade I were clustered step by step in the *Bougainvillea* genus with strong support (BS = 99-100%, and PP = 0.99-1) (Fig 9, S2 Fig).

The authors clearly failed to address all four major issues. For major #2, the significancy in including the thornless mutant was not well explained and elaborated.

Response: Thanks for your idea. The thornless mutant was a variety from *Bougainvillea × buttiana* 'Miss Manila'. The mother line of *Bougainvillea × buttiana* 'Miss Manila' has been sold out in commercial activities so far. However, the bud mutant has been kept in live and cultivated by grafting. Because the author He-Fa Wang hasn't yet got certificate of registered commercial name for the mutant. In the manuscript, we gave the mutant with the name of *Bougainvillea × buttiana* 'Miss Manila' sp.1.

For major #4, the breeding techniques do not affect the presentation of SNPs and Indels which are significant in cultivar authentication. It is understandable their are plenty of SNPs discovered from the analysis. Yet, the authors failed the listed out those valuable in cultivar authentication in an reader-friendly manner.

Response: Thanks for your idea. We didn't use the presentation of SNP and indels as described in cultivars of *Hyacinthus orientalis*. For three comparisons, there were more than 700 SNPs and 100 indels. This situation is not fit in the text. However, we listed out these SNPs and indels in detail information in the supplementary file: S7 Table, including base information of SNPs, mutant type, gene name, gene start, gene end, indel sequence, indel start, indel end, alignment strand and so on.

For major #1 and #3, the authors should obtain more suggestions froms experienced taxonomists and phylogenists, respectively. A numbers of minor issues were also not well addressed. The authors should read more articles to find out which format "the xxx genus/family" or "the genus/family xxx" should be correct in English.

Response: Thanks for your idea. These two forms have appeared in many articles. We selected the form of "the XXX genus/family" in the text.

The authors also failed to add authorities after genus name and species epithets, which are commonly adopted in the scientific research articles.

Response: The name of 12 *Bougainvillea* cultivar were adopted in the scientific book as following.

Liu Y, Ruan L, Zhou H, Yu M. Cultivar classification of *Bougainvillea*. China Forestry Press, Beijing, China, 2020

Sun L. Molecular identification of cultivars and transcriptome analysis of bracts in *Bougainvillea*. Ph. D. Chinese Academy of Forestry, Beijing, China, 2019, pp38-39

The presentation of GPS coordinates was in wrong order.

Response: We checked and revised the correct GPS in correct order. We reported details about the GPS for sample collection, DNA sample store and the rest leaf materials store.

Fresh leaves of twelve *Bougainvillea* cultivars, namely, *B.× buttiana* 'Mahara' , *B. × buttiana* 'Gautama's Red', *B. × buttiana* 'California Gold', *B. × buttiana* 'Double Salmon', *B. × buttiana* 'Double Yellow', *B. × buttiana* 'Big Chitra', *B. × buttiana* 'Los Banos Beauty', *B. glabra* 'White Stripe', *B. spectabilis* 'Flame', *B. spectabilis* 'Splendens', *B. × buttiana* 'Barbara Karst', and *B. × buttiana* 'San Diego Red' (Fig 1, S1 Table), were collected from the Provincial Flower Germplasm Resources Bank of San Jiao Mei in Zhangzhou (117°37′47″E, 24°28′35″N), Fujian Province, China. One bud mutation armed with simple or no thorns and derived from *B. × buttiana* 'Miss Manila', given namely, *B. × buttiana* 'Miss Manila' sp. 1 (Fig 1, S1 Table), was collected from the

cultivation factoty of Zhangzhou (117°49′9″E, 24°31′33″N) in Xiamen Qianrihong Horticulture Co., Ltd, Fujian Province, China. Fresh leaves were quickly frozen on dry ice, sent to the laboratory of the Environmental Horticulture Research Institute (113°20′39″E, 23°8′51″N) at the Guangdong Academy of Agricultural Sciences, Guangzhou, China, and stored at −80 °C until use. Genomic chloroplast DNA was extracted from each sample using the modified sucrose gradient centrifugation method [13]. Then, the DNA quality and quantity were checked through agarose gel electrophoresis and the NanoDrop microspectrometer method, respectively. Each qualified DNA sample was sheared to fragments of approximately 350 bp. Short-insert (350 bp) paired-end libraries were constructed, and sequencing was performed on an Illumina NovaSeq 6000 platform with a paired read length of 150 bp (Biozeron, Shanghai, China). The raw data from each sample were checked using FastQC v. 0.11.9 (http://www.bioinformatics.babraham.ac.uk/projects/fastqc/), and adaptors and low-quality reads were subsequently deleted by Trimmomatic v. 0.39 [14] with default parameters. The remaining materials, including the leaves and DNA, were deposited in the laboratory of the Environmental Horticulture Research Institute (store sheet code: B2023, 113°20′39″E, 23°8′51″N), Guangdong Academy of Agricultural Sciences, Guangzhou, China, as vouchers (S1 Table).

Suggestions on figure and table presentation were sadly ignored.

Response: We revised Figure 1 to Figure 9 as following.

We did not ignore the presentation of Table 4. For table 4, ‘462 L 0.969’ and ‘462 |L|0.969’ were two different forms for presentation. We use the form of ‘462 L 0.969’, not used the form of ‘462 |L|0.969’. Because the analyzed result from the CodeML program used the form of ‘462 L 0.969’, not used the form of ‘462 |L|0.969’.

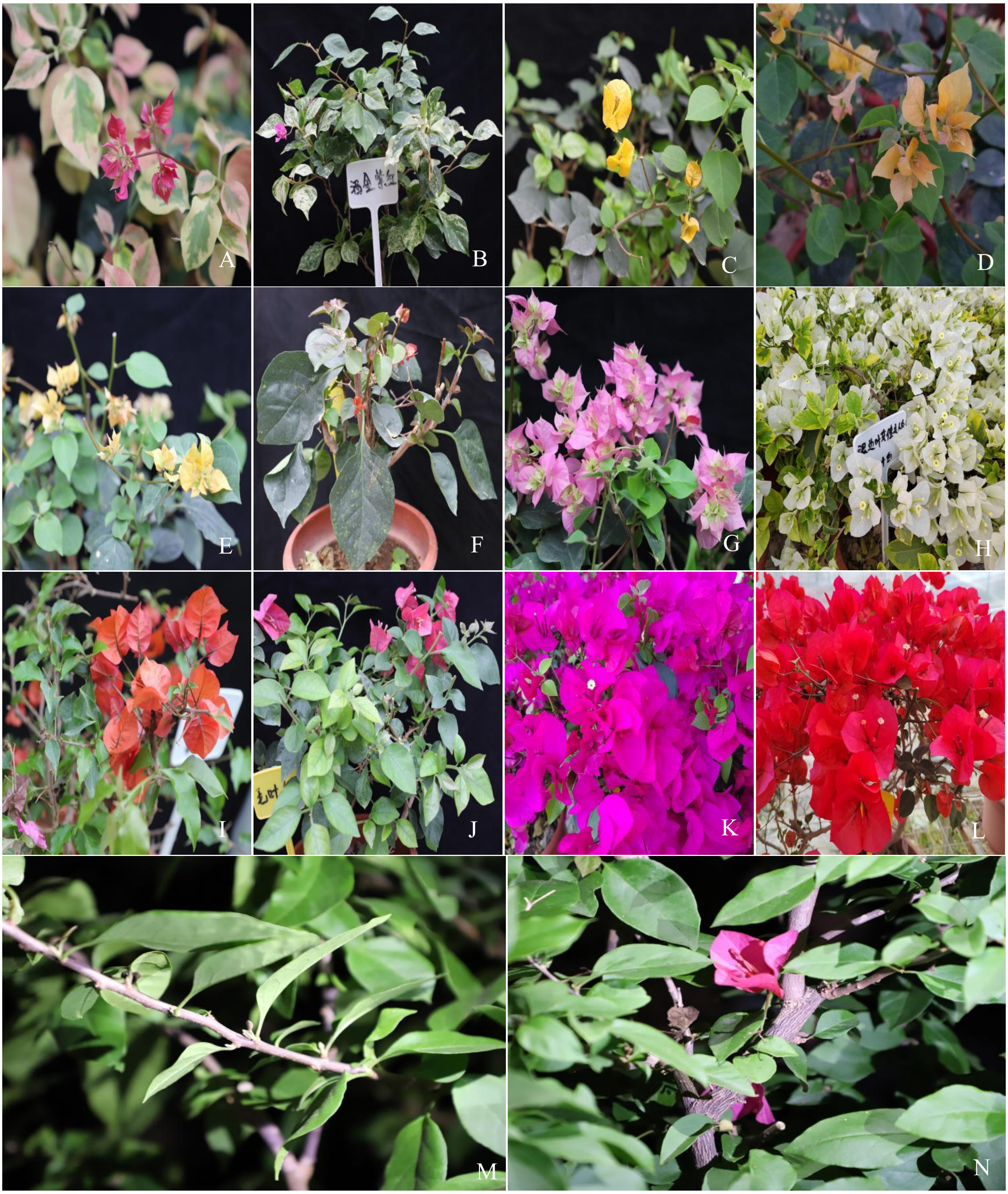

Fig 1. Morphologies among 13 cultivars of the *Bougainvillea* genus. A, *Bougainvillea×buttiana* 'Mahara'; B, *B.×buttiana* 'Gautama's Red'; C, *B. ×buttiana* 'California Gold'; D, *B.×buttiana* 'Double Salmon'; E, *B.×buttiana* 'Double Yellow'; F, *B. ×buttiana* 'Big Chitra'; G, *B.×buttiana* 'Los Banos Beauty'; H, *Bougainvillea glabra* 'White Stripe'; I, *Bougainvillea spectabilis* 'Flame'; J, *B. spectabilis* 'Splendens'; K, *B. × buttiana* 'Barbara Karst'; L, *B. × buttiana* 'San Diego Red'; M and N, *B. × buttiana* 'Miss Manila' sp. 1. armed with simple or no thorns, derived from *B. × buttiana* 'Miss Manila'.

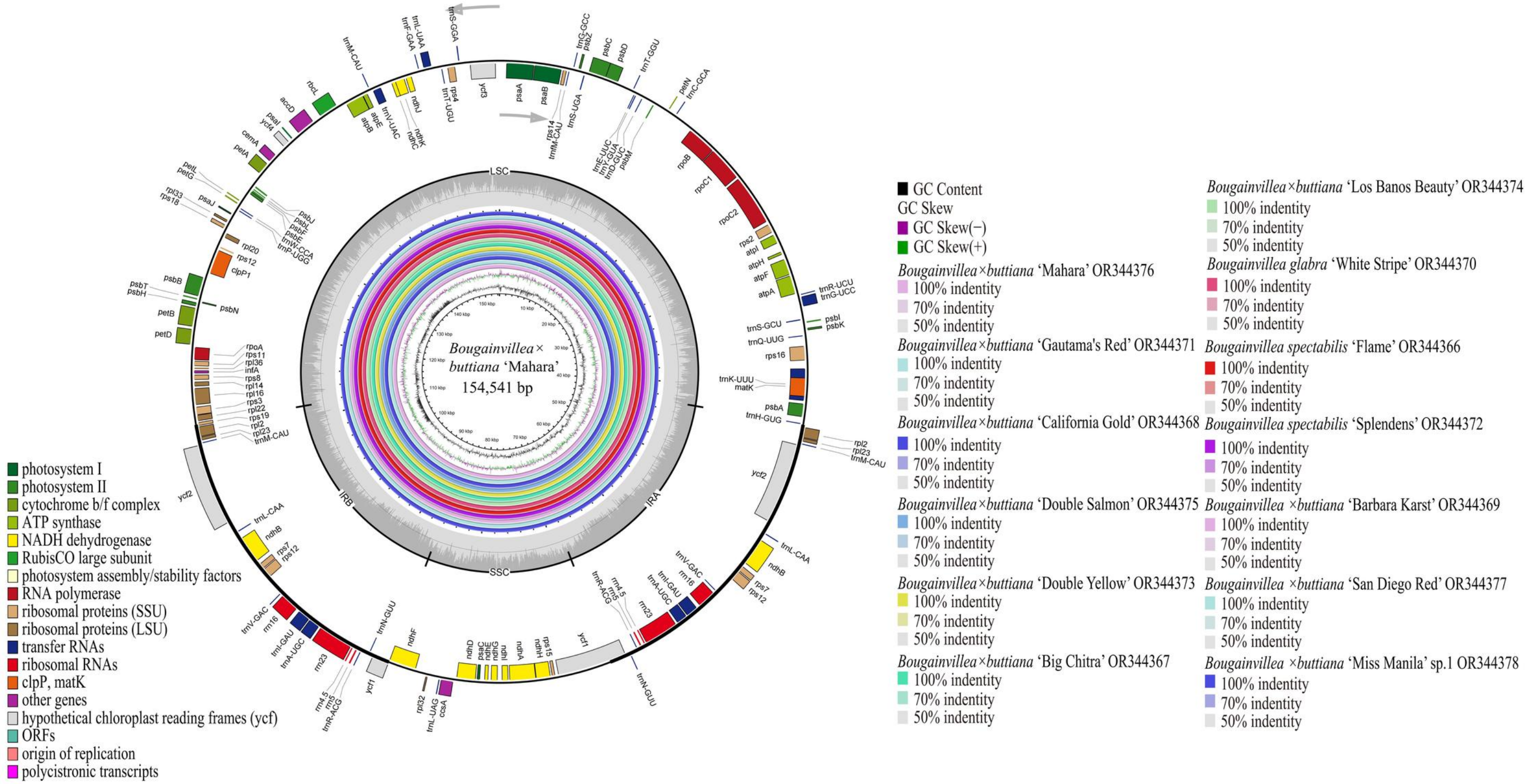

Fig 2. Chloroplast genome map of *B.* × *buttiana* 'Mahara' (the outermost three rings) and CGView comparison of 13 complete chloroplast genomes of *Bougainvillea* cultivars (the inner rings with different colors). Genes shown on the outside of the outermost first ring are transcribed counter-clockwise, and those on the inside are transcribed clockwise. The second ring with the darker gray color corresponds to the GC content, whereas the third ring with the lighter gray color corresponds to the AT content of the *B.* × *buttiana* 'Mahara' chloroplast genome generated by OGDRAW. The gray arrowheads indicate the directions of the genes. LSC, large single -copy region; IR, inverted repeat; SSC, small single-copy region. The innermost first black ring indicates the chloroplast genome size of *B.* × *buttiana* 'Mahara'. The innermost second and third rings indicate deviations in the GC content and GC skew, respectively, in the chloroplast genome of *C. barbatus*: GC skew + indicates G > C, and GC skew − indicates G < C. CGView comparison of the 13 complete chloroplast genomes of *Bougainvillea* cultivars displayed from the innermost 4th colored ring to the outer 16th ring: *B.* × *buttiana* 'Mahara', *B.* × *buttiana* 'Gautama's Red', *B.* × *buttiana* 'California Gold', *B.* × *buttiana* 'Double Salmon', *B.* × *buttiana* 'Double Yellow', *B.* × *buttiana* 'Big Chitra', *B.* × *buttiana* 'Los Banos Beauty', *B. glabra* 'White Stripe', *B. spectabilis* 'Flame', *B. spectabilis* 'Splendens', *B.*× *buttiana* 'Barbara Karst', *B.*× *buttiana* 'San Diego Red', and *B.* × *buttiana* 'Miss Manila' sp. 1, respectively. Chloroplast genome similar and highly divergent locations are represented by continuous and interrupted track lines, respectively.

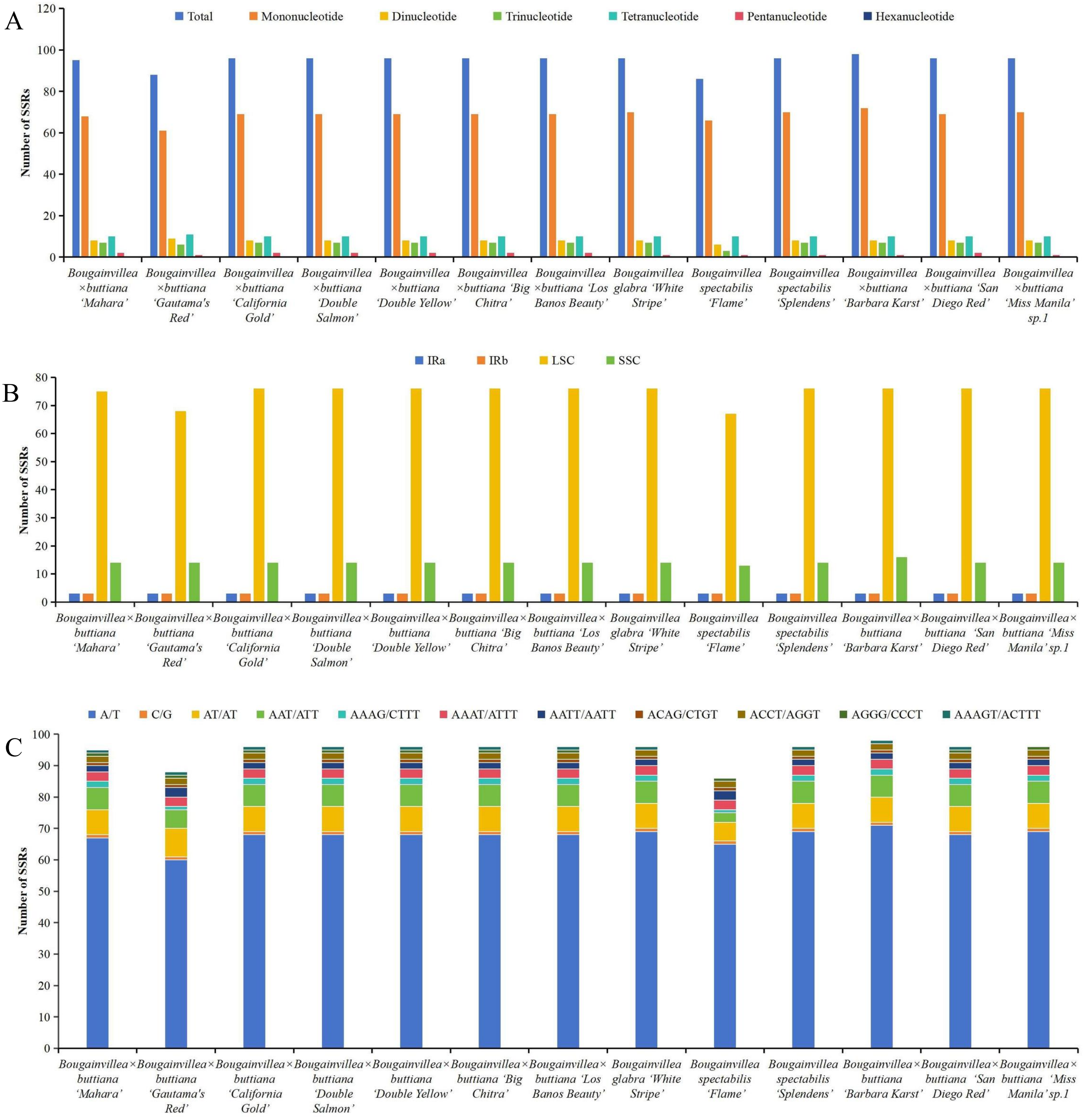

Fig 3. Distribution of SSRs in the 13 newly sequenced *Bougainvillea* chloroplast genomes. (A) Numbers of different SSR types detected in the 13 chloroplast genomes. (B) Frequencies of SSRs in the LSC, IR and SSC regions. (C) Frequencies of identified SSR motifs in different repeat class types.

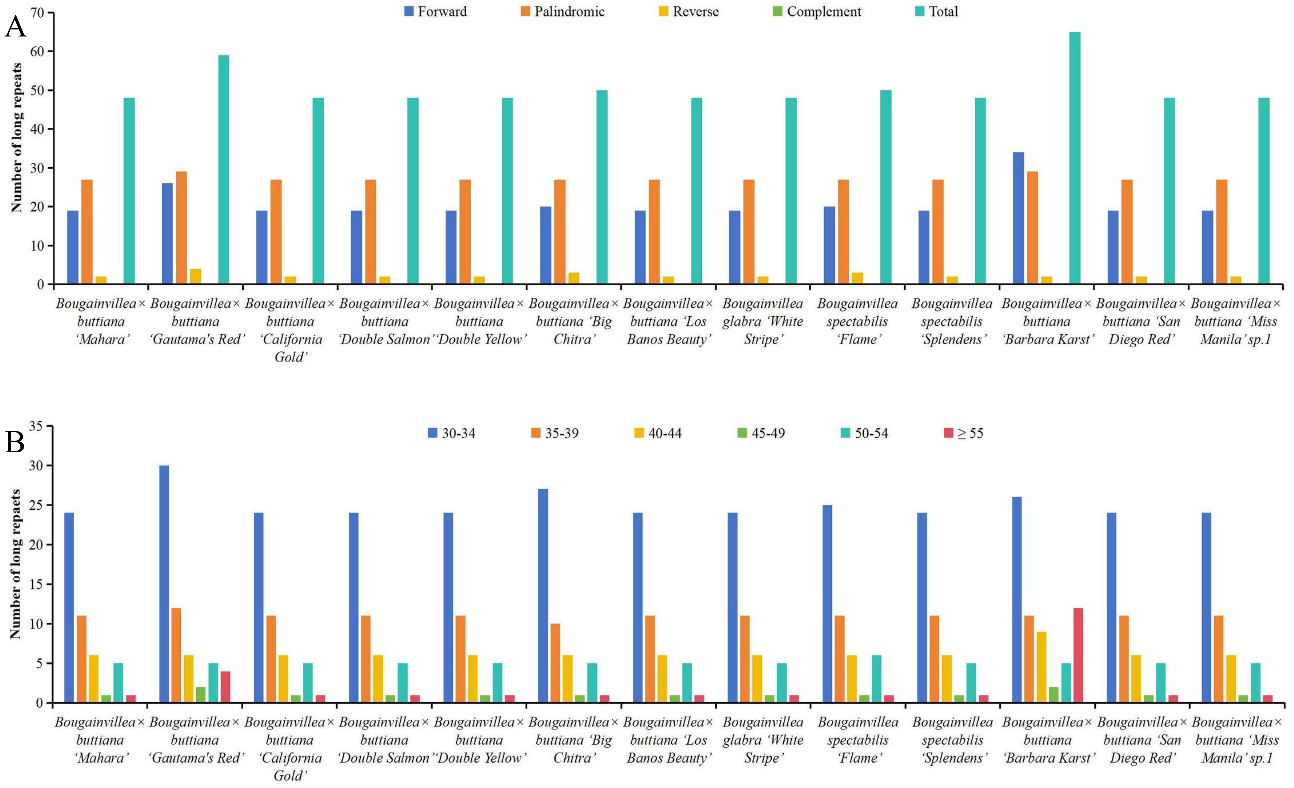

Fig 4. Analysis of long repeat sequences in the 13 newly sequenced *Bougainvillea* chloroplast genomes. (A) Total numbers of four long repeat types. (B) Length distribution of long repeats in each sequenced chloroplast genome.

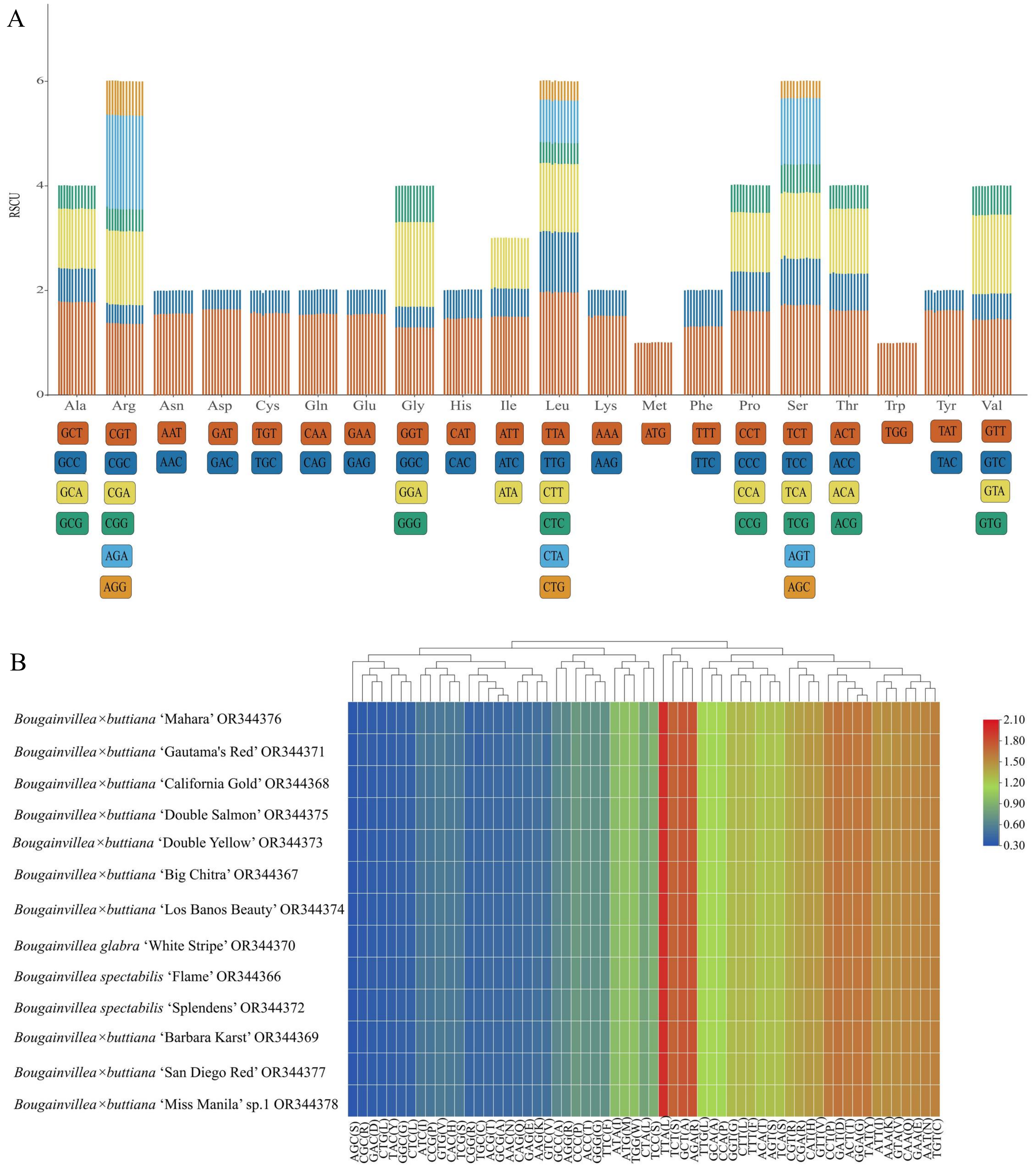

Fig 5. Codon contents of all protein-coding genes of 13 newly sequenced complete chloroplast genomes of the *Bougainvillea* cultivars. (A) Codon content and codon usage of the 20 amino acids and stop codons of all protein-coding genes. Each histogram from left to right is shown for *B.* × *buttiana* ‘Mahara’, *B.* × *buttiana* ‘Gautama's Red’, *B.* × *buttiana* ‘California Gold’, *B.* × *buttiana* ‘Double Salmon’, *B.* × *buttiana* ‘Double Yellow’, *B.* × *buttiana* ‘Big Chitra’, *B.* × *buttiana* ‘Los Banos Beauty’, *B. glabra* ‘White Stripe’, *B. spectabilis* ‘Flame’, *B. spectabilis* ‘Splendens’, *B.*× *buttiana* ‘Barbara Karst’, *B.*× *buttiana* ‘San Diego Red’, and *B.* × *buttiana* ‘Miss Manila’ sp. 1, respectively. (B) Heatmap analysis of the codon distribution of all protein-coding genes in the 13 newly sequenced chloroplast genomes.

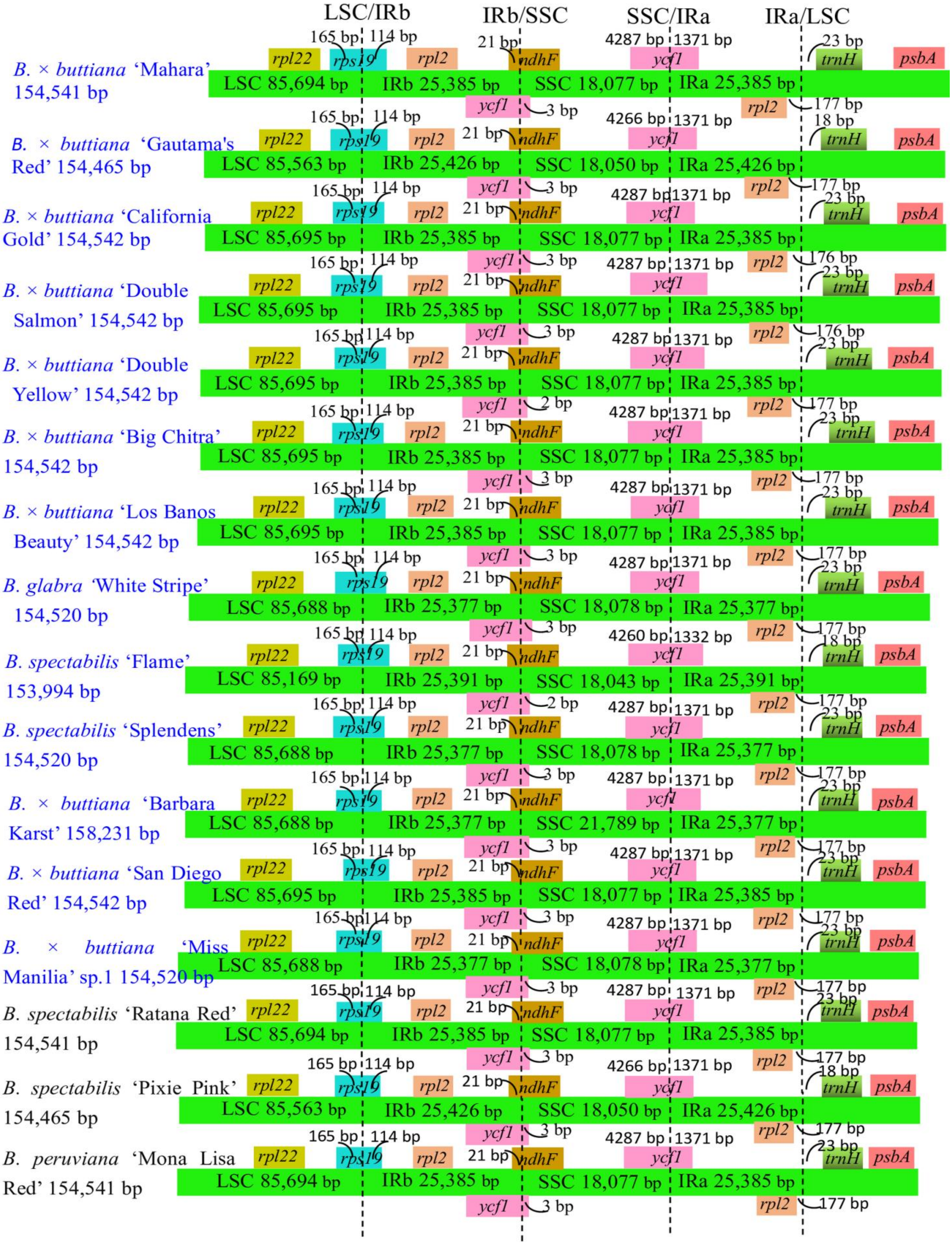

Fig 6. Comparison of the borders of the LSC, SSC, and IR regions among the 16 *Bougainvillea* chloroplast genomes. The 13 newly sequenced *Bougainvillea* chloroplast genomes identified in this study are shown in blue.

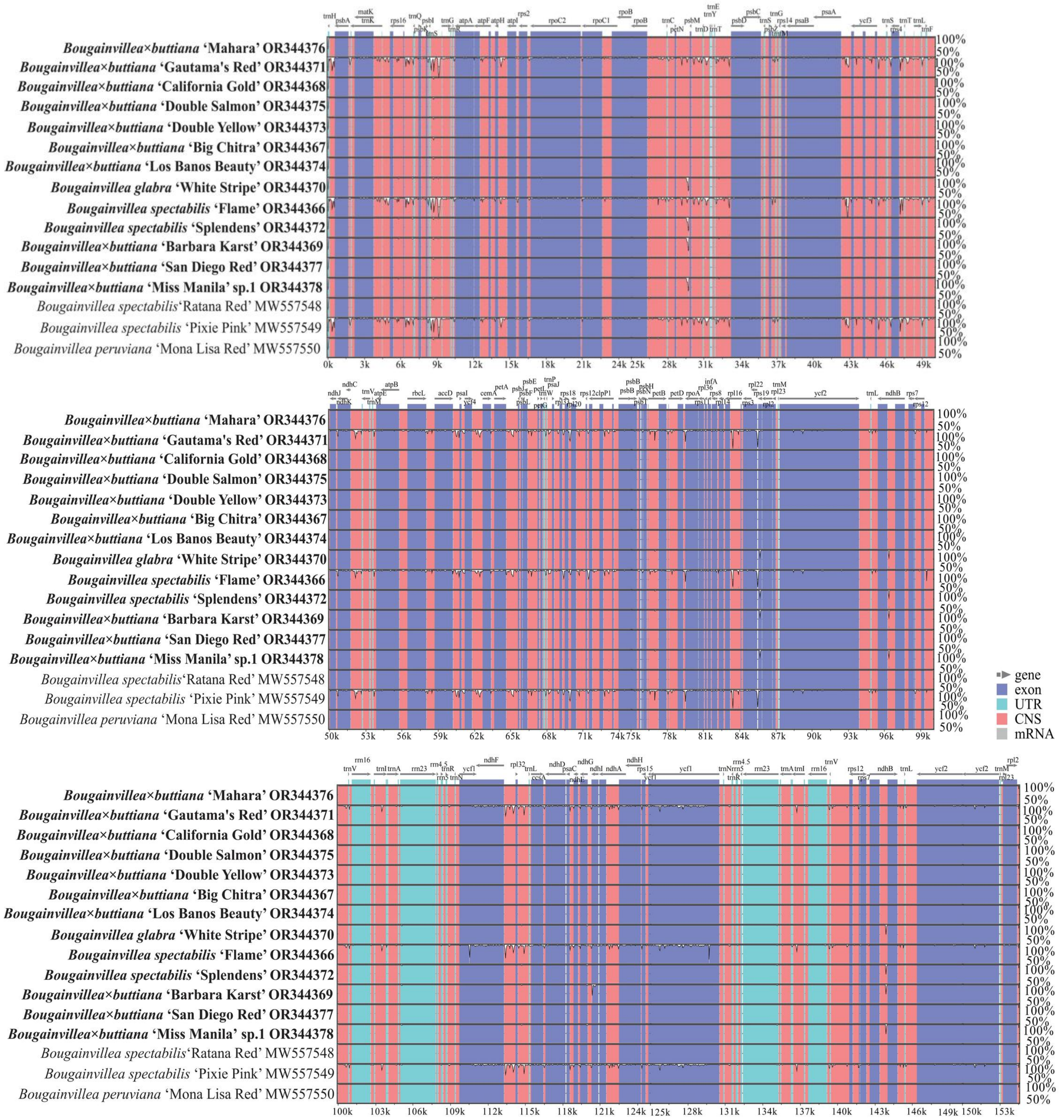

Fig 7. Complete chloroplast genome comparison of the 16 *Bougainvillea* chloroplast genomes using *B.* × *buttiana* 'Mahara' as a reference. The gray arrows and thick black lines above the alignment indicate gene orientation. Purple bars represent exons, sky-blue bars represent untranslated regions (UTRs), red bars represent non-coding sequences (CNS), gray bars represent mRNAs, and white regions represent sequence differences among the analyzed chloroplast genomes. The y-axis represents the identity percentage ranging from 50% to 100%. The 13 sequenced *Bougainvillea* chloroplast genomes in this study are shown in bold.

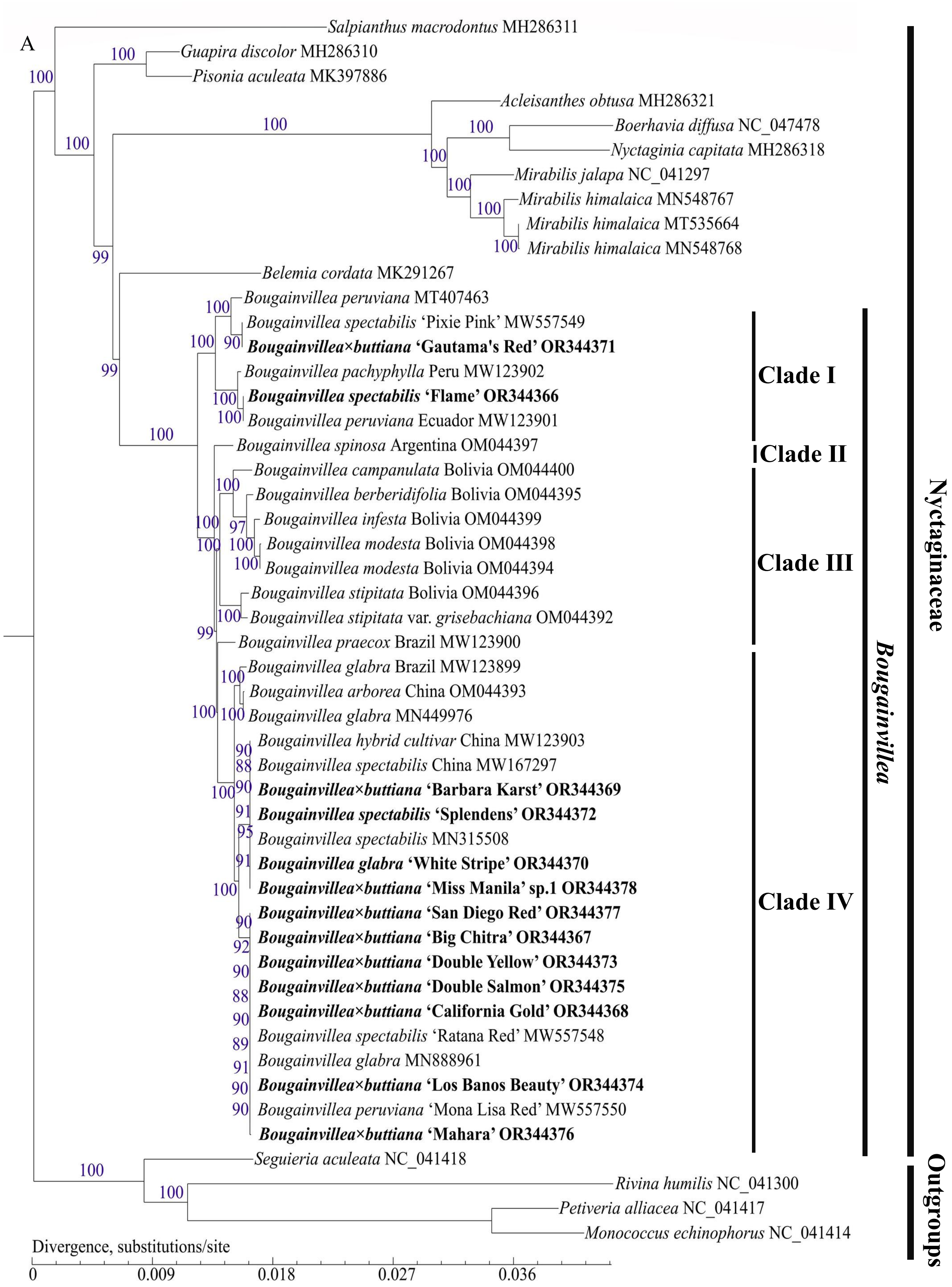

Fig 9. Phylogenetic relationships of Nyctaginaceae species based on chloroplast genomes sequences reconstructed using maximum likelihood (ML) and Bayesian inference (BI) methods. (A) ML tree. (B) BI tree. The 13 newly sequenced *Bougainvillea* chloroplast genomes identified in this study are shown in bold.

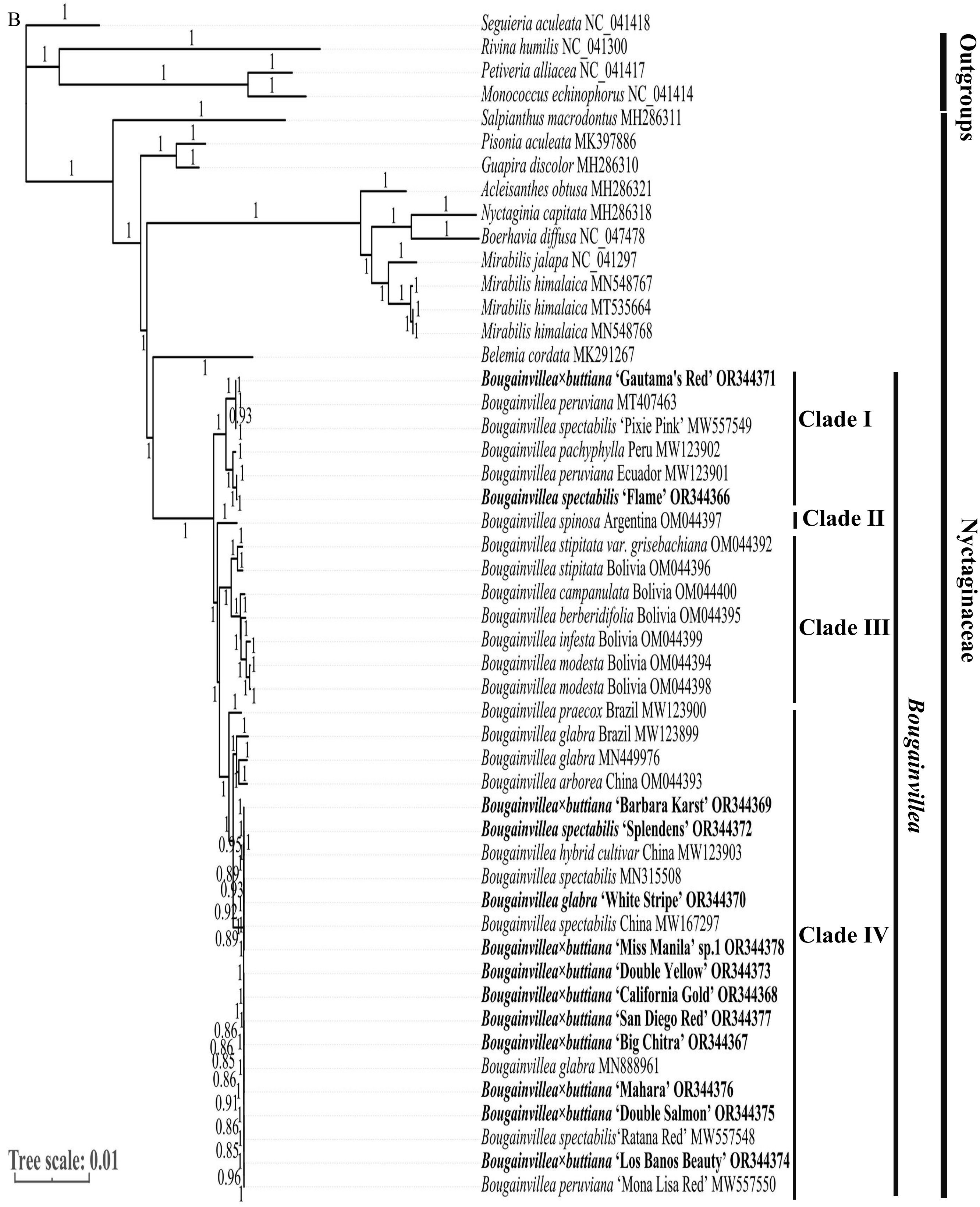

Fig 9. continued.

Reviewer #3: The overall write up is convincing. As this work is on the characterization of Bougainvillea cultivars, it should not be related to anything about taxonomy. Eventually, I have two concerns for this manuscripts:

1. There are a number of published work on the complete chloroplast genome of Bougainvillea. Considering that all information are important, I would be looking at a discussion on the difference of findings between this work and other published works, and the reasons for the difference identified in these analyses. For example, Lin et al. 2023, International Journal of Molecular Science 24:15138 should be the latest work before this work. I neither see it cited, nor being discussed. Lin et al. had similar analyses when compared to the current one. Please make sure all published work on the complete chloroplast genome of Bougainvillea are included in this work and compared for their findings.

Response: Thank you for this idea. We cited the article (Lin et al. 2023), compared and discussed the chloroplast genome, phylogenetic tree and related results.

Lin X, Lee SY, Ni J, Zhang X, Hu X, Zou P, Wang W, Liu G. Comparative analyses of chloroplast genome provide effective molecular markers for species and cultivar identification in *Bougainvillea*. Int J Mol Sci. 2023; 24(20),15138. https://doi.org/10.3390/ijms242015138. PMID: 37894819

2. Since some of the cultivars are named along with their parent (perhaps they are mutant clones), it would be reasonable to at least investigate the intraspecific genetic distance of these cultivar derived from the (presumably) same species. At least the analysis is conducted on the species assembled in this study, i.e., *B. x buttiana* and *B. spectabilis*.

Response: This is a good question. There were two main reasons that we did not sequence and assemble the wild species of *B.×buttiana* and *B. spectabilis*. First, wild species of *B. spectabilis* had been sequenced and reported in previous studies before, such as in Wang et al. 2019 and Bautista et al. 2020, 2022. Second, in the Provincial Flower Germplasm Resources Bank of San Jiao Mei in Zhangzhou (117°37′47″E, 24°28′35″N), Fujian Province, China, there are so many cultivars of *B.×buttiana*. However, the wild species of *B.×buttiana* was not easy to be obtained and identified. Therefore, we used the published chloroplast genomes of *B. spectabilis* in NCBI for phylogenetic analysis.

Based on this suggestion, we added the SNPs/indels analyses among groups of *B.×buttiana* ‘Mahara’ vs. *B. spectabilis* ‘Flame’, *B.×buttiana* ‘Mahara’ vs. *B. spectabilis* ‘Splendens’, and *B. spectabilis* ‘Splendens’-OR253994 (Lin et al. 2023) vs. *B. spectabilis* ‘Splendens’-OR344372 (this study). The details results can be found in two sections of SNPs and indels analyses among the

thirteen complete chloroplast genomes, and Intraspecific analyses of two chloroplast genomes of *B. spectabilis* ‘Splendens’.

Bautista MAC, Zheng Y, Boufford DE, Hu Z, Deng Y, Chen T. Phylogeny and taxonomic synopsis of the genus Bougainvillea (Nyctaginaceae). Plants (Basel). 2022; 11, 1700. https://doi.org/10.3390/plants11131700 PMID: 35807654

Bautista MAC, Zheng Y, Hu Z, Deng Y, Chen T. Comparative analysis of complete chloroplast genome sequences of wild and cultivated *Bougainvillea* (Nyctaginaceae). Plants (Basel). 2020; 9, 1671. https://doi.org/10.3390/plants9121671 PMID: 33260641

Lin X, Lee SY, Ni J, Zhang X, Hu X, Zou P, Wang W, Liu G. Comparative analyses of chloroplast genome provide effective molecular markers for species and cultivar identification in *Bougainvillea*. Int J Mol Sci. 2023; 24(20),15138. https://doi.org/10.3390/ijms242015138. PMID: 37894819

Wang N, Qiu MY, Yang Y, Li JW, Zou XX. Complete chloroplast genome sequence of *Bougainvillea spectabilis* (Nyctaginaceae). Mitochondrial DNA B Resour. 2019; 4, 4010-4011. https://doi.org/ 10.1080/23802359.2019.1688716 PMID: 33366293

Reviewer #4: The manuscript titled 'Comparative analysis of the complete chloroplast genomes of thirteen Bougainvillea cultivars from South China with implications for their genome structures and phylogenetic relationships' presents the assembly and annotation of 13 cp genomes of Bougainvillea. The study is well-organized and offers valuable insights for analyzing genetic diversity and phylogenetic relationships within the Nyctaginaceae family. I highly recommend this work for publication in the PLoS One journal.

I think the following minor suggestions and comments will help the authors improve their work.

1. The authors should provide additional information of the origin of the bud mutation, such as which plant accession did this mutation derive from?

Response: Thank you for this idea. The thornless mutant was a variety from *Bougainvillea* × *buttiana* ‘Miss Manila’. The mother line of *B.* × *buttiana* ‘Miss Manila’ has been sold out in commercial activities so far. However, the bud mutant has been kept in live and cultivated by grafting. Because the author He-Fa Wang hasn’t yet got certificate of registered commercial name for the mutant. In the manuscript, we gave the mutant with the name of *B.* × *buttiana* ‘Miss Manila’ sp. 1.

2. I wondered why these authors constructed so many types of phylogenetic trees, including two ML and BI trees based on whole cp genome sequences and coding sequences. These authors should

point out what's the difference between ML and BI trees, and which sequence is more appropriate to construct phylogenetic tree.

Response: Thank you for this idea. We thought that both the chloroplast genome sequences data and protein-coding genes sequences data were very important. Based on the four phylogenetic trees, the phylogenetic trees constructed by complete chloroplast genome sequences data were better.

In the section **Phylogenetic relationships,** we reported the differences between ML and BI trees as following:

Four phylogenetic trees were constructed using chloroplast genome sequences and protein-coding genes via the ML and BI methods, respectively (Fig 9, S2 Fig). Four species of Petiveriaceae were used as outgroups. The ML and BI trees from complete chloroplast genomes and protein-coding genes showed similar topological structures within 9 genera of Nyctaginaceae and differed in support values and positions among several *Bougainvillea* cultivars (Fig 9, S2 Fig). The 9 genera within Nyctaginaceae, *Bougainvillea*, *Belemia*, *Mirabilis*, *Nyctaginia*, *Boerhavia*, *Acleisanthes*, *Pisonia*, *Guapira* and *Salpianthus*, were strongly supported based on complete chloroplast genomes and protein-coding genes (bootstrap values, BS = 98-100% for the ML trees and posterior probabilities, PP = 1 for the BI trees) (Fig 9, S2 Fig).

Within Nyctaginaceae, *Bougainvillea* was strongly supported as a sister to *Belemia* (BS = 98–99% for the ML trees and PP = 1 for the BI trees) (Fig 9, S2 Fig). The 35 *Bougainvillea* individuals analyzed were divided into four clades, namely, Clades I, II, III, and IV, with strongly supported values (BS = 85–100% for the ML trees and PP = 0.99–1 for the BI trees) (Fig 9, S2 Fig). Two cultivars, *B.* × *buttiana* 'Gautama's Red' and *B. spectabilis* 'Flame', were clustered into clade I, and the other 11 cultivars, including *B.* × *buttiana* 'Mahara', *B.* × *buttiana* 'California Gold', *B.* × *buttiana* 'Double Salmon', *B.* × *buttiana* 'Double Yellow', *B.* × *buttiana* 'Los Banos Beauty', *B.* × *buttiana* 'Big Chitra', *B.* × *buttiana* 'Barbara Karst', *B. glabra* 'White Stripe', *B. spectabilis* 'Splendens', *B.* × *buttiana* 'San Diego Red', and *B.* × *buttiana* 'Miss Manila' sp. 1, were clustered into clade IV (Fig 9, S2 Fig). In Clade I, *B. spectabilis* 'Flame' was sister to *B. peruviana*

MW123901 and then formed a strong sister cluster to *B. pachyphylla*, both based on chloroplast genome sequences and protein-coding genes (Fig 9, S2 Fig). However, the position of *B. × buttiana* 'Gautama's Red' in the ML tree constructed from chloroplast genome sequences differed from those in the other three phylogenetic trees in this study. For the former, *B. × buttiana* 'Gautama's Red' was sister to *B. spectabilis* 'Pixie Pink' and then clustered with *B. peruviana* MT407463 with strong support (BS = 90%) (Fig 9A). For the latter, *B. spectabilis* 'Pixie Pink' was sister to *B. peruviana* MT407463 and then clustered with *B. × buttiana* 'Gautama's Red' with strong support (BS = 93–100%, and PP = 0.93–1) (Fig 9B, S2 Fig). In Clade II, it only contained *B. spinosa* (Fig 9, S2 Fig). In Clade III, there were 7 individuals of wild species, including *B. campanulata*, *B. berberidifolia*, *B. infesta*, *B. modesta* OM44398, *B. modesta* OM044396, *B. stipitata*, and *B. stipitata var. grisebachiana*. In Clade IV, in the ML tree based on the chloroplast genome sequences, *B. × buttiana* 'Mahara', *B. × buttiana* 'California Gold', *B. × buttiana* 'Double Salmon', *B. × buttiana* 'Double Yellow', *B. × buttiana* 'Los Banos Beauty', *B. × buttiana* 'Big Chitra', *B. × buttiana* 'San Diego Red', *B. spectabilis* 'Ratana Red', *B. glabra* MN888961, and *B. peruviana* 'Mona Lisa Red' were clustered together in one cluster with strong support (BS = 88–92%). *B. × buttiana* 'Barbara Karst', *B. glabra* 'White Stripe', *B. spectabilis* 'Splendens', *B. × buttiana* 'Miss Manila' sp. 1, *B. spectabilis* MN315508, *B. spectabilis* China MW167297, and *B.* hybrid cultivar MW123903 were clustered together in another cluster with strong support (BS =88–95%) (Fig 9A). However, in the ML tree based on protein-coding genes, *B. × buttiana* 'Mahara' was sister to the other cultivars in Clade IV with strong support (BS = 100%) (S2A Fig). In both BI trees, *B. × buttiana* 'Mahara', *B. × buttiana* 'California Gold', *B. × buttiana* 'Double Salmon', *B. × buttiana* 'Double Yellow', *B. × buttiana* 'Los Banos Beauty', *B. × buttiana* 'Big Chitra', *B. × buttiana* 'San Diego Red', *B. spectabilis* 'Ratana Red', *B. glabra*, *B. peruviana* 'Mona Lisa Red', *B. × buttiana* 'Barbara Karst', *B. glabra* 'White Stripe', *B. spectabilis* 'Splendens', *B. × buttiana* 'Miss Manila' sp. 1, *B. spectabilis* MN315508, *B. spectabilis* MW167297, and *B.* hybrid cultivar MW123903 were clustered together in one cluster in Clade IV with moderate to strong support (PP = 0.84–1) (Fig 9B,

S2B Fig). In the four phylogenetic trees, Clades III and IV were clustered together, forming a cluster with strong support (BS =85–99%, and PP = 0.99–1); and then the cluster, Clade II, and Clade I were clustered step by step in the *Bougainvillea* genus with strong support (BS = 99-100%, and PP = 0.99-1) (Fig 9, S2 Fig).
