# Supplementary material for: Comparative analysis of the complete chloroplast genomes of thirteen Bougainvillea cultivars from South China with implications for their genome structures and phylogenetic relationships
Source: PLoS One. 2024 Sep 11;19(9):e0310091. doi: 10.1371/journal.pone.0310091 (PMC11389920; doi:10.1371/journal.pone.0310091)
Supplement: Attachment — Submitted filename: PONE-D-23-37508-point-by-point response.docx [file pone.0310091.s012.docx]

PONE-D-23-37508
Characterization and comparative analysis of the complete chloroplast genomes of thirteen Bougainvillea cultivars with implications for their genome structures and phylogenetic relationships
PLOS ONE
Dear Dr. Li,
Thank you for submitting your manuscript to PLOS ONE. After careful consideration, we have decided that your manuscript does not meet our criteria for publication and must therefore be rejected.
Specifically:

Authors have not done justice to the problem which they have identified in this manuscript. The overall writing of the manuscript is very week specially the discussion. The authors completely failed in addressing the issues which they have raised in the manuscript. I will recommend a reject for this manuscript at this point of time.
I am sorry that we cannot be more positive on this occasion, but hope that you appreciate the reasons for this decision.

Kind regards,

Pankaj Bhardwaj, Ph.D.
Academic Editor
PLOS ONE

Authors have not done justice to the problem which they have identified in this manuscript. The overall writing of the manuscript is very week specially the discussion. The authors completely failed in addressing the issues which they have raised in the manuscript.

Response: We rewrote the manuscript PONE-D-23-37508 and revised the title, short title, abstract, introduction, results, deleting Figure 5 in the text, and discussion. The places in the text where were revised by us using red in the form of PONE-D-23-37508 track changes.
