# Supplementary material for: Comparative analysis of the complete chloroplast genomes of thirteen Bougainvillea cultivars from South China with implications for their genome structures and phylogenetic relationships
Source: PLoS One. 2024 Sep 11;19(9):e0310091. doi: 10.1371/journal.pone.0310091 (PMC11389920; doi:10.1371/journal.pone.0310091)
Supplement: Attachment — Submitted filename: response to reviewers.docx [file pone.0310091.s013.docx]

Reviewers' comments:

Reviewer's Responses to Questions

**Comments to the Author**

1. Is the manuscript technically sound, and do the data support the conclusions?

The manuscript must describe a technically sound piece of scientific research with data that supports the conclusions. Experiments must have been conducted rigorously, with appropriate controls, replication, and sample sizes. The conclusions must be drawn appropriately based on the data presented.

Reviewer #1: Partly

Reviewer #2: Yes

Response: We revised the whole text and conclusion carefully using red markers.

2. Has the statistical analysis been performed appropriately and rigorously?

Reviewer #1: Yes

Reviewer #2: Yes

3. Have the authors made all data underlying the findings in their manuscript fully available?

The [PLOS Data policy](http://www.plosone.org/static/policies.action#sharing) requires authors to make all data underlying the findings described in their manuscript fully available without restriction, with rare exception (please refer to the Data Availability Statement in the manuscript PDF file). The data should be provided as part of the manuscript or its supporting information, or deposited to a public repository. For example, in addition to summary statistics, the data points behind means, medians and variance measures should be available. If there are restrictions on publicly sharing data—e.g. participant privacy or use of data from a third party—those must be specified.

Reviewer #1: Yes

Reviewer #2: Yes

4. Is the manuscript presented in an intelligible fashion and written in standard English?

PLOS ONE does not copyedit accepted manuscripts, so the language in submitted articles must be clear, correct, and unambiguous. Any typographical or grammatical errors should be corrected at revision, so please note any specific errors here.

Reviewer #1: No

Reviewer #2: Yes

Response: We revised the English of the text carefully using red markers by American Journal Experts (AJE). We believe that the revised manuscript is more readable.

5. Review Comments to the Author

Please use the space provided to explain your answers to the questions above. You may also include additional comments for the author, including concerns about dual publication, research ethics, or publication ethics. (Please upload your review as an attachment if it exceeds 20,000 characters)

Reviewer #1: The study of Wu et al. domenstrates the application of chloroplast genomes in explore the germplasm of Bougainvillea cultivars. Apart from the phylogenetic relationships between the cultivars, potential molecular markers including SSRs, LSRs and hotspot regions have been investigated from the chloroplast genomes. The study shows merits on the research of Bougainvillea, that could serve as a reference in studying other horticultural crops with diversified morphologies. However, prior to a make a further decision, the following major and minor issues should be resolved.

Major issues:
1) The cultivars of Bougainvillea are regulated by the International Code for the Nomenclature for Cultivated Plants (ICNCP). The registration of Bougainvillea cultivars is designated to the Indian Agricultural Research Institute (IARI). The authors are responsible to carefully check if the cultivar epithets are well established and registered. Illegitimate epithets (e.g. misspelling and homonyms) and unestablished epithets are common in horticultural germplasms. The authors should refer to the Articles 25 to 27 of ICNCP (9th Edition) which is available online (https://www.ishs.org/sites/default/files/static/ScriptaHorticulturae_18.pdf). The publications of The Bougainvillea Society of India (BSI) of IARI (http://www.bsi-iari.com/publication.htm) will help the authors in checking. The authors should indicate those unestablished and unregistered cultivars in the manuscript as precaution for the readers.
Response: Thank you for this question. We can’t open the BSI of IARI. So the cultivars in the manuscript were unregistered in this web. We wrote in Data Availability Statement as following: The 13 *Bougainvillea* cultivars studied in this study are unregistered in the *Bougainvillea* Society of India (BSI) of IARI (http://www.bsi-iari.com/publication.htm).

2) I wonder if the mutant (Bougainvillea sp.1) is discovered by the authors themselves?

Response: Thank you for this question. Yes, the mutant (*Bougainvillea* sp.1) was discovered by the author He-Fa Wang.

If so, what is the original cultivar of this mutant?

Response: The mutant (*Bougainvillea* sp.1) was from the other cultivar (mother line), of which the bract colour looked like the bract colour of the cultivar *Bougainvillea* ‘Barbara Karst’. This mother line has thorns and was sold out in commercial activities. However, the bud mutant was kept in live and grown by grafting. Because the author He-Fa Wang hasn’t yet got certificate of registered commercial name for the mutant. In the manuscript, we gave the mutant with the name of *Bougainvillea* sp.1.

The authors should elaborate why this mutant is included in this study.

Response: In this study, we want to know the phylogenetic relationships among the mutant (*Bougainvillea* sp.1) and other individuals of the *Bougainvillea* genus by using complete chloroplast genomes.

Also, Figure S1 (the images of 13 cultivars) should be included in the main text, prior to the chloroplast genome map. Vouchers of the studied cultivars, either live and dried specimens, should be listed out with their collection location, GPS, date of collection, collector numbers, and deposited herbarium or institution.
Response: Thank you for this question. We revised Figure 1 instead Figure S1 in the manuscript. In the Plant materials, DNA extraction, and sequencing section, we wrote detail information of the studied cultivars, either live and dried specimens, with their collection location, GPS, date of collection, collector numbers, and deposited herbarium or institution.
Fresh leaves of twelve *Bougainvillea* cultivars and one bud mutation, namely, *B.*× buttiana ‘Mahara’ , B. × buttiana ‘Gautama's Red’, B. × buttiana ‘California Gold’, B. × buttiana ‘Double Salmon’, B. × buttiana ‘Double Yellow’, B. × buttiana ‘Big Chitra’, B. × buttiana ‘Los Banos Beauty’, *B.* glabra ‘White Stripe’, *B.* spectabilis ‘Flame’, *B.* spectabilis ‘Splendens’, *B.* ‘Barbara Karst’, *B.* ‘San Diego Red’, and *B.* sp. 1 (Fig 1, S1 Table), were collected from the Provincial Flower Germplasm Resources Bank of San Jiao Mei (117°37′47″E, 24°28′35″N) in Zhangzhou, Fujian Province, China. Fresh leaves were quickly frozen on dry ice, sent to the laboratory of the Environmental Horticulture Research Institute at the Guangdong Academy of Agricultural Sciences (113°21′8″E, 23°9′2″N), Guangzhou, China, and stored at −80 ℃ until use. Genomic chloroplast DNA was extracted from each sample using the modified sucrose gradient centrifugation method [13]. Then, the DNA quality and quantity were checked through agarose gel electrophoresis and the NanoDrop microspectrometer method, respectively. Each qualified DNA sample was sheared to fragments of approximately 350 bp. Short-insert (350 bp) paired-end libraries were constructed, and sequencing was performed on an Illumina NovaSeq 6000 platform with a paired read length of 150 bp (Biozeron, Shanghai, China). The raw data from each sample were checked using FastQC v. 0.11.9 (http://www.bioinformatics.babraham.ac.uk/projects/fastqc/), and adaptors and low-quality reads were subsequently deleted by Trimmomatic v. 0.39 [14] with default parameters. The remaining materials, including the leaves and DNA, were deposited in the laboratory of the Environmental Horticulture Research Institute (store sheet code: B2023), Guangdong Academy of Agricultural Sciences, Guangzhou, China, as vouchers (S1 Table).

**Fig 1.** Morphologies among 13 cultivars of the *Bougainvillea* genus. A, *Bougainvillea glabra ‘*White Stripe’; B, *Bougainvillea*×*buttiana* ‘Mahara’; C, *Bougainvillea*×*buttiana* ‘Double Yellow’; D, *Bougainvillea*×*buttiana* ‘Double Salmon’; E, *Bougainvillea*×*buttiana* ‘Los Banos Beauty’; F, *Bougainvillea* ×*buttiana* ‘California Gold’; G, *Bougainvillea* ×*buttiana* ‘Big Chitra’; H, *Bougainvillea*×*buttiana* ‘Gautama's Red’; I, *Bougainvillea spectabilis* ‘Flame’; J, *Bougainvillea spectabilis* ‘Splendens’; K, *Bougainvillea* ‘Barbara Karst’; L, *Bougainvillea* ‘San Diego Red’; M and N, *Bougainvillea* sp1. armed with simple or no thorns.

3) I noticed that *Bougainvillea spinosa* was included in Clade II by the authors. However, it should not be regarded as a member of Clade II, as it is sister to all other members from both clade II and III (in both Figure 8 A&B and S3 A&B). The authors should discuss the potential reasons in the Discussion.
Response: Thank you for this question. *Bougainvillea spinosa* should be clustered in Clade Ⅰ. We revised the results of phylogenetic relationships as following.

In Clade Ⅰ, B. spectabilis ‘Flame’ was sister to *B. peruviana* MW123901, then forming a cluster strongly sister to *B. pachyphylla*, both based on chloroplast genome sequences and protein-coding genes (Fig 9, S2 Fig). However, the position of B. *×* buttiana ‘Gautama's Red’ in ML tree constructed by chloroplast genomes sequences was different from the other three phylogenetic trees in this study. For the former, B. *×* buttiana ‘Gautama's Red’ was sister to *B. spectabilis* ‘Pixie Pink’, then clustered with *B. peruviana* MT407463 with strong support (BS = 90%) (Fig 9A). For the latter, *B. spectabilis* ‘Pixie Pink’ was sister to *B. peruviana* MT407463, then clustered with B. *×* buttiana ‘Gautama's Red’ with strong support (BS = 93-100%, and PP = 0.93-1) (Fig 9B, S2 Fig). Then, *B. spinosa* and these 6 individuals were clustered together with strong support in Clade Ⅰ (Fig 9, S2 Fig). In Clade Ⅱ, there were 7 individuals of wild species, including *B. campanulata*, *B. berberidifolia*, *B. infesta*, *B. modesta* OM44398, *B. modesta* OM044396, *B. stipitata*, and *B. stipitata var. grisebachiana*.

4) The definition of SNPs and InDels in this manuscript should be well defined. Could the nucleotide differences between one and another cultivar be considered as SNPs and InDels?

Molecular Diagnostic Characters (MDCs) of a cultivar to differentiate itself from the other 12 cultivars could be more meaningful. Also, haplotype analysis could aid in visualizing the figures in this tables by grouping the cultivars. The authors could refer to the following article:
Wong, K. H., Wu, H. Y., Kong, B. L. H., But, G. W. C., Siu, T. Y., Hui, J. H. L., Shaw, P. C., & Lau, D. T. W. (2022). Characterisation of the complete chloroplast genomes of seven *Hyacinthus orientalis* L. cultivars: Insights into cultivar phylogeny. Horticulturae, 8(5), 1-28.
Response: Thank you for this question. We didn’t use the presentation of SNP and indels as described in cultivars of *Hyacinthus orientalis.* For one reason, in my opinion, the background of *Hyacinthus orientalis* was less complicated than the background of the cultivars of *Bougainvillea*. The breeding of *Hyacinthus orientalis* didn’t use grafting; however, grafting is very common in cultivation of *Bougainvillea* cultivars. For other reason, too many SNPs and indels (more than 100), such as *B. glabra ‘*White Stripe’ vs. B. *×* buttiana ‘Gautama's Red’, *B. glabra ‘*White Stripe’ vs. B. spectabilis ‘Flame’ and *B. ×* buttiana ‘Mahara’ vs. B. *×* buttiana ‘Gautama's Red’, were not suitable in the text. We also showed the SNPs and indels of different comparisons in the supplementary file (S7 Table).

Minor issues:
The writing style and language of the manuscript, particularly the abstracts, introduction and the discussion, should be meticulously improved. I have identified a number of mistakes in grammar and use of wordings which deteriorate the comprehensibility of the manuscript. In addition, I highly recommend the authors to employ a native English speaker or professional editing agency to proofread the manuscript. The style in presenting figures and tables should also be improved. Please kindly refer to the following:
L16-18: Having high similarity in leaf appearance and hybridization among Bougainvillea species, the phylogenetic relationships of the genus are complicated and controversial.
Response: Thank you for this idea. We revised as following:

Having high similarity in leaf appearance and hybridization among *Bougainvillea* species, especially from Bougainvillea × buttiana, their phylogenetic relationships were very complicated and controversial.

L20-21: Their phylogenetic relationships within the genus Bougainvillea and other species of the family Nyctaginaceae are identified for the first time.
Response: Thank you for this idea. We revised the sentence as following:

In this study, we sequenced, assembled and analyzed thirteen complete chloroplast genomes of *Bougainvillea* cultivars from South China, including seven B. × buttiana cultivars and six other Bougainvillea cultivars, and identified their phylogenetic relationships within the Bougainvillea genus and other species of the Nyctaginaceae family for the first time.

L27-28: Four divergent regions, including ......, were identified from sliding window analysis of 16 Bougainvillea cultivar genomes.
Response: Thank you for this idea. Comparative analysis not only contained sliding window analysis (mVISTA), but also contained CGView analysis and nuclear diversity analysis (Pi). Therefore, we revised the sentence as following:

Four divergent regions, including *trnH-GUG_psbA*, *trnS-GCU_trnG-UCC-exon1*, *trnS-GGA_rps4*, and *ccsA_ndhD*, were identified from comparative analysis of 16 Bougainvillea cultivars genomes.

L36: replace "which contained" by "including"
Response: Thank you for this idea. We revised ‘including’ instead of ‘which contained’.

L39: ..., but also helped to identify Bougainvillea
Response: Thank you for this idea. We revised ‘helped to identify *Bougainvillea*’

L51: "Le Du Juan", which is well-known in China, belongs to the genus Bougainvillea of the family Nyctaginaceae.
Response: Thank you for this idea. We revised as following:

“Le Du Juan”, which is well-known in China, belongs to the *Bougainvillea* genus of the Nyctaginaceae family.

L53-54: with colored bracts [2,3]. The colorful bracts surrounding the small tubular flowers are often mistakenly treated as flowers.
Response: Thank you for this idea. We revised as following:

The colorful bracts surrounding the small tubular flowers are often mistakenly treated as flowers.

L59: remove "value"
Response: Thank you for this idea. We removed ‘value’.

L60: replace "only" by "mainly"
Response: Thank you for this idea. We revised ‘mainly’ instead of ‘only’.

L60-61: ... challenging because of high similarity
Response: Thank you for this idea. We revised ‘challenging because of high similarity’.

L68: replace "identified by" by "explored using"
Response: Thank you for this idea. We revised ‘explored using’ instead of ‘identified by’.

L69: Why is this sentence concerning the usage of Bougainvillea is placed here? Recommend placing it in the previous paragraphs.
Response: Thank you for this idea. We placed this sentence in the previous paragraph.

L70: delete "that has been"
Response: Thank you for this idea. We deleted ‘that has been’.

L71: replace "taken" by "introduce"
Response: Thank you for this idea. We revised ‘introduce’ instead of ‘taken’.

L75: ... B. x buttiana cultivars and the molecular evolution ...
Response: Thank you for this idea. We revised as following:

However, phylogenetic relationships of B. × buttiana cultivars and molecular evolution of chloroplast genomes from the Nyctaginaceae family still remains to be unveiled [2-5,9-12].

L77-78: In this study, complete chloroplast genomes of thirteen Bougaivillea cultivars were newly sequence, assembled and annotated. These thirteen cultivars from South China include seven ....
Response: Thank you for this idea. We revised as following:

In this study, complete chloroplast genomes of thirteen Bougainvillea cultivars were newly sequenced, assembled and annotated. These thirteen cultivars from South China included seven B. × buttiana cultivars,

L84: ... integrating three published ...
Response: Thank you for this idea. We revised as following:

by integrating three published complete chloroplast genomes

L85: Bougainvillea cultivars on the NCBI. In this study, five objectives were targeted: (1) ... [Aim =/= objectives!]
Response: Thank you for this idea. We revised as following:

In this study, five objectives were targeted

L97: please state the full name of the "resource garden" here.
Response: Thank you for this idea. We wrote the full name as following:

Provincial Flower Germplasm Resources Bank of “San Jiao Mei” (117°37′47″E, 24°28′35″N) at Zhangzhou, Fujian Province, China.

L99: remove "finally"
Response: Thank you for this idea. We removed ‘finally’.

L101-102: ... the DNA quality and quantity was checked through ... method, respectively. ...
Response: Thank you for this idea. We revised as following:

Then the DNA quality and quantity was checked through agarose gel electrophoresis and nanodrop microspectrometer method, respectively.

L104: is the read in 150 bp paired or not???
Response: The read is in 150 bp paired.

L109: assembly and annotation were conducted using the methods previously reported [15].
Response: Thank you for this idea. We revised as following:

Chloroplast genome assembly and annotation were conducted using the methods previously reported [15].

L125: ... The long repeats were detected with a minimum ....
Response: Thank you for this idea. We revised as following:

The long repeats were detected with a minimum repeat size of 30 bp, a Hamming distance of 3, and a repeat identity of more than 90%.

L143: remove "ones"
Response: Thank you for this idea. We removed ‘ones’.

L163: Phylogenetic trees were constructed using ...
Response: Thank you for this idea. We revised as following:

Phylogenetic trees were constructed using Maximum likelihood (ML) and Bayes inference (BI) methods, respectively.

L186 and elsewhere throughout the manuscript: "different genes" should be better replaced as "unique genes".
Response: Thank you for this idea. ‘different genes’ means the genes with the different name, not the same name. We thought ‘different’ is better than ‘unique’. So we used ‘different’ in the manuscript.

L253 and the parts reporting IR: please keep consistency with the border titles with Fig. 5. For example, in L253 "IRb/LSC" should be replaced as "LSC/IRb".
Response: Thank you for this idea. We revised as following:

Comparison details of the LSC/IR and SSC/IR boundaries were performed among these 13 chloroplast genomes and 3 published chloroplast genomes of *Bougainvillea* cultivars (Fig 6)*.* Regarding LSC/IRb borders, the *rps19* gene was located at the boundaries of the LSC/IRb borders in all 16 *Bougainvillea* chloroplast genomes. The *rps19* gene expanded into the IRb regions with distances of 114 bp in all 16 *Bougainvillea* chloroplast genomes (Fig 6). Regarding IRa/LSC borders, the *rpl2* and *trnH-GUG* genes were located at the boundaries of the IRa/LSC borders in all 16 *Bougainvillea* chloroplast genomes. The distances between the ends of *rpl2* and IRa/LSC borders ranged from 176 bp to 177 bp (Fig 6). The distances between the ends of *trnH-GUG* and IRa/LSC borders ranged from 18 bp to 23 bp (Fig 6).

The SSC/IRa border was located in the *ycf1* region, which crossed into the IRa region in all 16 *Bougainvillea* chloroplast genomes, with distances ranging 1332 bp to 1371 bp, respectively (Fig 6). For the IRb/SSC borders, *ycf1* expanded into the SSC regions in all 16 *Bougainvillea* chloroplast genomes overlap with the *ndhF* gene. A total of 2 bp or 3 bp distance was identified between the end of *ycf1* and the IRb/SSC border and a 21 bp distance between the start of *ndhF* and the IRb/SSC border (Fig 6). Overall, the LSC/IR boundary regions of the 16 *Bougainvillea* chloroplast genomes were highly conserved, but the SSC/IR boundary regions exhibited slight variations.

L303 and elsewhere throughout the manuscript: the authority of genera and species should be shown for the first appearance in the manuscript. For example, Bougainvillea Comm. ex Juss., Belemia Pires, Mirabilis L. Latin should be italic.
Response: Thank you for this idea. The names of genera and species should be italic in the whole text.

L310-311: the authors could discuss more concerning the different phylogentic positions of the cultivars of B x buttiana 'Gautama's Red' and 'Mahara'. Would it suggest a different origin of them?
Response: Thank you for this idea. We discussed this situation in the discussion part as following:

In the present study, our four phylogenetic trees obtained from chloroplast genome sequences and protein-coding genes revealed that *Bougainvillea* was a sister to *Belemia* with strong support (BS = 98–99%, and PP = 1) (Fig 9, S2 Fig). This result was broadly consistent with those of previous studies [1,6]. We also found that 35 *Bougainvillea* individuals within the genus *Bougainvillea* were divided into three clades with strong support (Fig 9, S2 Fig). This finding was in agreement with a previous study [3]. However, the four phylogenetic trees in this study displayed some inconsistencies in the *Bougainvillea* genus, such as the shifting position of B. *×* buttiana ‘Gautama's Red’ in Clade Ⅰ (Fig 9, S2 Fig). However, more *Bougainvillea* cultivar chloroplast genomes may need to be sequenced to resolve their positions. Nonetheless, based on our phylogenetic results, we propose that B. *×* buttiana ‘Gautama's Red’ may be from *B. peruviana* and *B. spectabilis*. Additionally, the other eleven cultivars, including the remaining six B. × buttiana cultivars, were clustered in Clade III in the *Bougainvillea* genus (Fig 9, S2 Fig). Grafting techniques are often used in the cultivation processes of B. × buttiana cultivars, and based on the results of our four phylogenetic trees, we speculated that these seven B. × buttiana cultivars may come from hybrids involving *B. peruviana*, *B. spectabilis* and *B. glabra.* These results were, to some extent, in agreement with a previous hypothesis, which presumed that the B. × buttiana cultivars may be hybrids of B. peruviana and B. glabra [5].

L325: Why *B. glabra* 'White Stripe' is not clustered with other cultivars of *B. glabra*?
Response: Thank you for this idea. Although *B. glabra* 'White Stripe' was not clustered with other samples of *B. glabra*, *B. glabra* 'White Stripe' and other samples of *B. glabra* were all clustered in the clade III. This may because the genetic variation of the chloroplast genome of *B. glabra* 'White Stripe' was derived from its other parent during the processes of hybridization or grafting.

L374: replace "with" by "in"
Response: Thank you for this idea. We thought ‘with’ was better than ‘in’ here.

Fig 1. It might be meaningless to include the GCView Comparison results since they are too thin which are hardly can see. The layout of chloroplast genome map would be too complicated. The authors could consider removing the GC comparison from this figure and submit it as a supplementary figure.
Response: Thank you for this idea. GCView results should be included in the text. We only showed the overall comparison of the 13 complete chloroplast genomes of *Bougainvillea* cultivars, not for details genes. We thought that GCView results should show in the text and shouldn’t submit as a supplementary file.

Table 4: The authors should define what the numbers and alphabets represent in the table. It is recommended to rearrange the table in a more easy-understanding manner e.g. by dividing the items of a positively selected sites in columns:
Positively selected sites
Gene xx |x| xxx
ndhF 462 |L|0.969*
502 |A|0.960*
Response: Thank you for this idea. We thought that the form of ‘462 L 0.969*’ was better than the form of ‘462 |L|0.969*’. So we used the form of ‘462 L 0.969*’ in Table 4.

Reviewer #2: Dear authors
I have reviewed this study from the beginning to the end and I did not see any deficiency in terms of the main headings (introduction, material-method, conclusion and discussion). The methods were selected in accordance with the purpose of the study and applied correctly. The language of the article is simple and understandable. There are no stylistic errors. The main objectives of the study are: (1) to characterise and study the structures of 13 newly sequenced complete chloroplast genomes; (2) to detect variation in simple sequence repeats 87 (SSR), long repeats and codon usage among these 13 chloroplast genomes; (3) to identify highly variable 88 regions for potential development of DNA markers among Bougainvillea cultivars; (4) to understand the molecular 89 evolution of chloroplast genomes in the Nyctaginaceae family; and (5) to reveal the phylogenetic relationships 90 between Bougainvillea species and varieties and other species in the Nyctaginaceae family. Therefore, I would like to say that the study is acceptable in its present form. Sincerely.

Response: Thank you for your suggestions.
